# Modulating voltage-gated sodium channels to enhance differentiation and sensitize glioblastoma cells to chemotherapy

**DOI:** 10.1186/s12964-024-01819-z

**Published:** 2024-09-09

**Authors:** Francesca Giammello, Chiara Biella, Erica Cecilia Priori, Matilde Amat Di San Filippo, Roberta Leone, Francesca D’Ambrosio, Martina Paterno’, Giulia Cassioli, Antea Minetti, Francesca Macchi, Cristina Spalletti, Ilaria Morella, Cristina Ruberti, Beatrice Tremonti, Federica Barbieri, Giuseppe Lombardi, Riccardo Brambilla, Tullio Florio, Rossella Galli, Paola Rossi, Federico Brandalise

**Affiliations:** 1 Department of Biology and Biotechnology “L. Spallanzani”, University of Pavia, Pavia, 27100 Italy; 2 IRCCS San Raffaele Hospital, Via Olgettina 58, Milan, 20132 Italy; 3 Department of Biosciences, University of Milan, Milan, 20133 Italy; 4 CNR Neuroscience Institute of Pisa, Via Giuseppe Moruzzi, 1, Pisa (PI), 56124 Italy; 5 Advanced Technology Platform, Department of Biosciences, University of Milan, Milan, 20133 Italy; 6 Pharmacology Unit, Department of Internal Medicine, University of Genova, Genova, 16132 Italy; 7 Department of Oncology 1, Oncology, Veneto Institute of Oncology IOV-IRCCS, via Gattamelata 64, Padua, 35128 Italy; 8 PhD Program in Genetics, Molecular and Cellular Biology, University of Pavia, Pavia, Italy; 9 IRCCS Ospedale Policlinico San Martino, Genova, 16132 Italy

**Keywords:** GBM, Na_v_, CANCER STEM CELLS, TMZ, TTX; CHEMOTHERAPY, ERK, MAPK, RESTING MEMBRANE POTENTIAL

## Abstract

**Background:**

Glioblastoma (GBM) stands as the most prevalent and aggressive form of adult gliomas. Despite the implementation of intensive therapeutic approaches involving surgery, radiation, and chemotherapy, Glioblastoma Stem Cells contribute to tumor recurrence and poor prognosis. The induction of Glioblastoma Stem Cells differentiation by manipulating the transcriptional machinery has emerged as a promising strategy for GBM treatment. Here, we explored an innovative approach by investigating the role of the depolarized resting membrane potential (RMP) observed in patient-derived GBM sphereforming cell (GSCs), which allows them to maintain a stemness profile when they reside in the G0 phase of the cell cycle.

**Methods:**

We conducted molecular biology and electrophysiological experiments, both in vitro and in vivo, to examine the functional expression of the voltage-gated sodium channel (Na_v_) in GSCs, particularly focusing on its cell cycle-dependent functional expression. Na_v_ activity was pharmacologically manipulated, and its effects on GSCs behavior were assessed by live imaging cell cycle analysis, self-renewal assays, and chemosensitivity assays. Mechanistic insights into the role of Na_v_ in regulating GBM stemness were investigated through pathway analysis in vitro and through tumor proliferation assay in vivo.

**Results:**

We demonstrated that Na_v_ is functionally expressed by GSCs mainly during the G0 phase of the cell cycle, suggesting its pivotal role in modulating the RMP. The pharmacological blockade of Na_v_ made GBM cells more susceptible to temozolomide (TMZ), a standard drug for this type of tumor, by inducing cell cycle re-entry from G0 phase to G1/S transition. Additionally, inhibition of Na_v_ substantially influenced the self-renewal and multipotency features of GSCs, concomitantly enhancing their degree of differentiation. Finally, our data suggested that Na_v_ positively regulates GBM stemness by depolarizing the RMP and suppressing the ERK signaling pathway. Of note, in vivo proliferation assessment confirmed the increased susceptibility to TMZ following pharmacological blockade of Na_v_.

**Conclusions:**

This insight positions Na_v_ as a promising prognostic biomarker and therapeutic target for GBM patients, particularly in conjunction with temozolomide treatment.

**Supplementary Information:**

The online version contains supplementary material available at 10.1186/s12964-024-01819-z.

## Background

Glioblastoma (GBM) is the deadliest tumor of the central nervous system (CNS), for which no effective cure currently exists [1, 2]. The poor prognosis of GBM arises not only from its high rate of aggressiveness and brain invasion but also from therapy resistance and tumor recurrence [3]. The standard of care for glioblastoma treatment involves surgical resection followed by localized radiation therapy and concomitant chemotherapy with temozolomide (TMZ) [4, 5].

Intratumoral heterogeneity is a major contributor to therapy failure [6]. Among the various cellular subtypes, patient-derived GBM sphere-forming cell (GSCs) drive tumor resistance and recurrence [7]. GSCs constitute a population of GBM cells with self-renewal and pluripotency abilities [8]. Current research is focusing on different approaches to target GSCs and improve therapeutic effectiveness against GBM. Cancer cells employ various strategies to escape radio- and chemotherapy-induced cell death [9], with the regulation of the cell cycle playing a crucial role. GSCs can disable checkpoints, which are activated when DNA damage occurs, leading to delayed or arrested cell cycle progression at G1/S, S, early G2, or late G2 checkpoints. GSCs, however, tend to stay predominantly in G0 [10]. Standard therapy primarily targets proliferative cells during G1/S transition, thus minimally affecting GSCs and allowing relapses at the end of treatments [11]. Current treatments aim to induce differentiation of GSCs through the modulation of transcription factors. However, a less explored additional mechanism influencing cell cycle transitions is the modulation of the resting membrane potential (RMP) [12], which sustains a polarized state. GSCs exhibit a depolarized RMP that correlates with a maintained stemness profile [13]. RMP plays a crucial role in cell differentiation and proliferation during development [14, 15], serving as a hallmark of sustained tumor growth in cancer [16]. The oscillation of RMP between hyperpolarization (in the G1/S transition) and depolarization (in the G2/M and G0 phase) results from the fine-tuned up- or down-regulation of key ion channels permeable to potassium, chloride, and, more recently highlighted, sodium [17]. Sodium dynamics have gained attention in other solid tumors such as prostatic, colorectal, and breast cancers [18]. Channels permeable to sodium (Na^+^) have been correlated with cancer cell invasion and metastasis [19]. However, little is known about their role in GBM. Previous studies reported that the acid-sensing ion channel 1a (ASIC1a), a voltage-independent channel, negatively modulates glioma stemness [20, 21]. Yet, there is a profound lack of investigation on the role of voltage-gated sodium channels (VGSCs). Voltage-gated sodium channels are composed of the pore-forming α subunit and the non-pore-forming β subunit. The α subunit forms the ion-conducting pore, while the β subunits modulate the gating properties of the channel and interact with various cellular signaling pathways. The β subunits are involved in regulating channel opening frequency and recruiting downstream effectors, contributing to the overall function and regulation of the VGSC complex [22]. Notably, clinical investigations have indicated a clear correlation between the expression of voltage-gated sodium channels (Na_v_) and a shorter life expectancy in GBM patients [23], yet the causal link between Na_v_ expression and GBM resistance and aggressiveness remains elusive. In this study, we elucidate how the Na_v_ channel functions as a gate for GSCs stemness, maintaining the RMP in a depolarized state. We reveal that Na_v_ channel negatively regulates both ERK1/2 and Akt downstream pathways, promoting GSCs persistence in the G0 phase and the expression of functional stemness markers such as SOX2 and NANOG. Our findings suggest that the modulation of Na_v_ and downstream signaling may serve as a potential therapeutic target, forcing GSCs into a proliferative and terminally differentiated state, thereby enhancing their susceptibility to gold standard glioblastoma therapy.

## Methods

### Human GSCs

Human primary GBM culture enriched in stem cells (GSCs) were generated in the laboratories of Professor T. Florio at the University of Genoa (Genoa, Italy) (GBM3, GBM19) from neurosurgical specimens after patients’ informed consent and Institutional Ethical Committee approval (CER Liguria number 360/2019) [24], and Dr. R. Galli (PhD) at San Raffaele University (Milan, Italy) (L0605, L0627, L1312). These GBM cultures were generated in compliance with the Declaration of Helsinki and with policies approved by the Ethics Boards of Spedali Civili di Brescia, University of Brescia (Italian Data Protection Authority Resolution #52, 24/7/2008 and DL 193/2003). The GSCs were isolated and maintained as previously described [25]. In brief, cells derived from patient GBM biopsy samples were plated at clonal density [26] in serum-free medium, containing EGF and FGF2 to sort away differentiated/differentiating cells, allowing neural stem cells to expand and proliferate [27].

Twenty to 40 days after plating, clones resembling the classical neurospheres were detected (early passages 10–15). Vials from this original batch had been stored in liquid nitrogen and defrost for each experiment. These cell lines were assessed for long-term proliferation, self-renewal, multipotency and tumorigenicity. GSCs from Florio’s laboratory were cultivated in suspension as spheroid aggregates in stem cell-permissive medium (1:1 DMEM High Glucose and Ham’s F-12 Nutrient Mix-GlutaMAX™ - Gibco™, Thermo Fisher Scientific), enriched with B27 supplement (Gibco™, Thermo Fisher Scientific), 10 ng/ml human basic fibroblast growth factor (bFGF, Miltenyi Biotec), and 20 ng/ml human epidermal growth factor (EGF, Miltenyi Biotec). The neurospheres were mechanically dissociated into a single-cell suspension. GSCs from the Galli’s laboratory were cultured in suspension using NeuroCult™ Basal Medium supplemented with NeuroCult™ Proliferation Supplement (STEMCELL Technologies). Upon GSCs neurospheres formation, they were collected, centrifuged, and the pellet was mechanically dissociated to obtain a single-cell suspension, which was then plated to allow for the propagation of the cell lines.

To provide comprehensive clinical annotations for these patient-derived GSCs, we have included a table below detailing the age, sex, mutation status, and IDH status of the samples (Table 1).

**Table 1:** Clinical annotations for the patient-derived GSCs investigated

Patients’ and tumors’ features									
Code	Sex	Age (yrs)	Location	Primary/ Recur.	WHO grade	Molecular Subtype	IDH and 1p/19q co-deletional status	NOD/SCID mice survival time (days)	Ki67 (%)
GBM3	M	48	N/A	*P*	IV	Proneural	N/A	120	N/A
GBM19	F	41	N/A	N/A	IV (secondary to oligodendroglioma)	Mesenchymal	N/A	100	N/A
L1312	N/A	59	Fronto-tempo-parietal	*P*	IV	Mesenchymal	N/A	N/A	35
L0627	N/A	N/A	N/A	*P*	IV	Classical	N/A	N/A	N/A
L0605	N/A	N/A	N/A	*P*	IV	Proneural	N/A	N/A	N/A

### WPMY-1 prostate myofibroblast

The WPMY-1 prostate myofibroblast cell line has been acquired from the American Type Tissue Culture Collection (ATCC, VA, USA). The DU145 and PC3 cell lines were cultured in RPMI-1640 medium (Lonza) with 10% FBS (Thermo Fisher Scientific) at 37 °C with 5% CO_2_. WPMY-1 cells were cultured in DMEM (Lonza) with 10% FBS (Gibco, Thermo Fisher Scientific Inc.).

### U87 human Glioblastoma cell line

U87 human Glioblastoma cell line was cultivated in adhesion in DMEM High Glucose (Sigma-Aldrich) with 10% FBS (Thermo Fisher Scientific) and 0.01% Pen/Strep and maintained at 37 °C in 5% O2 and 5% CO2. These cells were detached using trypsin-EDTA, collected, centrifuged and replated after reaching confluence.

### GL261 murine glioma cell line

GL261 murine glioma cell line, kindly provided by Dr. R. Galli, was cultivated in adhesion in DMEM High Glucose (Sigma-Aldrich) with 10% FBS (Thermo Fisher Scientific) and maintained at 37 °C in 5% O2 and 5% CO2. These cells were detached by scraper, collected, centrifuged and replated every 2 days.

### FUCCI plasmid mechanism of action

To generate GSCs expressing the Fluorescence Ubiquitin Cell Cycle Indicator (FUCCI) system stably, we acquired and utilized the pBOB-EF1-FastFUCCI-Puro plasmid (Addgene plasmid #86849). The FUCCI system comprises two fluorescent polypeptides that undergo ubiquitination and degradation by the proteasome in a cell cycle-dependent manner. FUCCI cells exhibit red fluorescence in the G1 phase, orange/yellow hue upon transitioning into S-phase, and green fluorescence in late S-phase. The green fluorescence persists through G2-phase and mitosis, where, during anaphase, the green probe undergoes degradation. Specifically, the FUCCI system leverages the phase-dependent characteristics of replication licensing factors Cdt1 and Geminin. A fusion protein of a fragment of Cdt1 (amino acids 30–120) with the fluorescent protein monomeric Kusabira-Orange 2 (mKO2) serves as an indicator of the G1 phase. Additionally, a fusion protein of a fragment of Geminin (amino acids 1-110 or 1–60) with the fluorescent protein monomeric Azami-Green 1 (mAG1) enables visualization of the S, G2, and M phases.

### Lentivirus production and infection

Plasmids encoding different portions of the viral structure, together with pBOB-EF1-FastFUCCI-Puro plasmid, were stably transfected in HEK293T cells using a Calcium Phosphate precipitation-based method. Once the lentivirus was produced, GSCs were plated in p35 Petri dishes to reach 50% confluence on the day of infection. Just before infection, Polybrene (Sigma-Aldrich, Cat. No. TR-1003) was added to the viral medium at a final concentration of 8 µg/ml. The cell growth medium was replaced by the viral one and incubated for 18–20 h. After incubation, the medium was substituted with a fresh one and the cells were checked under the fluorescence microscope to determine if they were already expressing the construct of interest. Cells have then been sorted for both green and red fluorescence positivity to select homogeneous and stable cell line.

### Cell plating and proliferation assay

Cells at T0 were plated in 24-wells multiwell plates, with at least 3 replicates for each time point (24, 48, 72, 96 h); after passaging and counting cells, 20,000 cells were plated in each well with 0,5 mL of fresh growth medium. For each time point, cells were collected from plates, centrifuged at 1,000 x g for 8 min and the pellet was resuspended in 90 µL of TryPLE™ Select (Thermo Fisher Scientific). Then, resuspended cells were counted using the following protocol: after being centrifuged and resuspended in new fresh growth medium, 25 µL of cells were collected and mixed in a 1:1 ratio with Trypan Blue 0,4% (Sigma-Aldrich) for the staining of dead cells; 10µL of the cells treated with Trypan Blue were positioned in glass Countess™ II FL Reusable Slides (Thermo Fisher Scientifics) to perform cell counting using the Countess™ II FL automated Cell counter (Thermo Fisher Scientifics). A growth curve comprising the average number of cells for each time point was then built.

For the experiments involving the TMZ (Sigma-Aldrich) 3 µM treatment for 288 h, fresh growth medium containing 3 µM TMZ was added every 72 h.

The TTX (HelloBio) 30 µM treatment was performed 72 h before starting the proliferation assay if not differently stated.

The following reagents were tested: temozolomide (Sigma-Aldrich), tetrodotoxin (HelloBio), riluzole (HelloBio), carbamazepine (Tocris Bioscience), rufinamide (HelloBio), ranolazine (Tocris Bioscience).

### Preparation of three-Dimensional (3D) GSCs cultures and proliferation progression assessment

Three-dimensional GSCs organoids were generated following established procedures [28]. In brief, a suspension of 5000 cells was prepared in ice-cold Matrigel™ and dispensed onto parafilm molds in 20µL droplets. The molds were then incubated at 37 °C for 30 min. Subsequently, the droplets were delicately detached from the molds and transferred to 6-well plates containing complete medium, followed by incubation in a 5% CO_2_. environment at 37 °C. The morphological development of organoids was monitored until dense aggregates formed, typically within 7–10 days.

To assess GSCs organoids progression, the multiwell was placed every day at the same time under an ImageXpress Micro Confocal system (Molecular Device). Z-stack acquisition was performed in a bright field configuration (picture acquisition every 160 μm). The surface of the GSCs organoids was assessed. The following conditions were applied: control (CTR), TTX 30µM, TMZ 3µM, TTX 30µM + RB5 25µM + TMZ 3µM, riluzole 20µM, riluzole 20µM + TMZ 3µM. All drugs were included 24 h before the acquisition.

At the end of the experiment the GSCs organoids were lysed after being detached from the multiwell through addition of Trypsin-EDTA 1X in PBS (Euroclone) to each well. Samples were incubated at 37 °C for 15 min and then centrifuged at 1,100 x g for 5 min at room temperature. The supernatant was removed, and the pellet was resuspended in PBS 1X, then a second centrifugation was performed at 1,100 x g for 5 min at room temperature. These following steps were performed in ice: the supernatant was removed, and the pellet was resuspended in cold RIPA buffer (Thermo Fisher Scientifics) added with proteases and phosphatases inhibitor cocktail 1X (Thermo Fisher Scientifics). Samples were vortexed every 10 min for 3 times. Samples were sonicated for 15 min intermittently and centrifuged at 13,000 x g for 15 min at 4 °C; finally, supernatants were collected.

### Clonogenic and neurosphere assay

The clonogenic assay (or colony formation assay) is an in vitro cell survival assay that measures the ability of single cells to grow into colonies. The reproductive capacity of cells (defined by the number and diameter of colonies) can be compared before and after specific treatments. Adhered cells were plated in 6-well plates, each well containing 300 cells in 2 mL of fresh growth medium. Cells were then incubated at 37 °C for a minimum of 10 days, to allow optimal growth of the colonies. The growth capacity of GSCs was thus compared between control condition and following 72 h pre-treatment with TTX 30 µM. After 11 days cells were fixed using ice-cold methanol and colonies were colored with Crystal Violet (CV) 0,1% (PanReac AppliChem) to evaluate the number and diameter of colonies obtained in different conditions. Colonies were counted and their diameter was quantified with respect to a reference measure unit using the ImageJ software in 8bit or 16bit format.

### MTT assay

GSCs cell viability was measured using the MTT [3-(4,5-dimethylthiazol-2-yl)-2,5-diphenyltetrazolium bromide] assay to determine whether the drugs applied to the cells were used at non-toxic concentrations. Briefly, cells were seeded in a 96-well plate at a density of 10,000 cells/well (0.2 mL medium per well) and incubated at 37 °C, 5% of CO_2,_ for 24 h. The following day, the culture medium was replaced with fresh medium added with the drug of interest. For the control condition, cells were incubated with only the culture medium. 72 h after exposure, 20 µL of MTT solution (HelloBio) was added to each well; this operation was performed in the dark, and the plates were subsequently incubated for 3 h at 37 °C. Then, cell viability was assessed by measuring the samples’ absorbance at 550 nm using the PerkinElmer Multimode Plate Reader (PerkinElmer Inc., Waltham, Massachussetts, USA).

### RT2 profiler PCR arrays test

According to the manufacturer’s instructions, total RNA was extracted from the GSCs using the Monarch Total RNA Miniprep Kit (Biolabs). RNA purity was measured by optical density and only samples with an OD 260/280 ratio ranging between 1.8 and 2 and an OD 260/230 greater or equal to 1.8 were used.

Reverse transcription was performed by using the All-in-One™ First-Strand cDNA Synthesis kit (GeneCopoeia, USA) under the following reaction conditions: 42 °C for 60 min and 70 °C for 5 min. The cDNA was used on the real-time RT2 Profiler PCR Array (QIAGEN, Cat. no. PAHS-176ZA-330231) in combination with RT2 SYBR^®^Green qPCR Mastermix (Cat. no. 330231). Each array plate contained one set of 96 wells for testing. Genomic DNA contamination, reverse transcription, and positive PCR controls were included in each 96-well set on each plate. Glyceraldehyde-3-phosphate-dehydrogenase (GAPDH) was used as the assay reference gene. Cycle threshold (CT) values were copied to an Excel file to build a table of CT values, which then was transferred onto the dedicated data analysis web portal at http://www.qiagen.com/geneglobe. Samples contained controls and test groups. CT values were normalized based on Automatic selection from a full panel of reference genes. The relative gene expression was calculated using the 2^−ΔΔ*CT*^ quantitative method and normalized with the housekeeping GAPDH. For the RT2 Profiler PCR Array the *p*-value was calculated based on a Student’s t-test. Changes in mRNA level for evaluated genes were assessed in all groups in relation to the control group.

### Whole cell patch clamp

Whole cell electrophysiological recordings were performed on patient-derived primary cultures enriched in GSCs to record Na_v_-mediated current. Cells were recorded while suspended, in a bath solution containing (in mM): NaCl 140, KCl 5, Hepes 10, glucose 5, CaCl_2_ 2, MgCl_2_ 1, pH 7.4. Glass borosilicate pipettes were filled with (in mM): KCl 135, NaCl 10, Hepes 10, MgCl_2_ 1, EGTA 1, CaCl_2_ 0.1, pH 7.2.

To isolate the Na_v_-mediated current in voltage clamp configuration, a series of voltage steps, 500ms long, were delivered to the cell, from − 70mV to + 120mV with an increment of 10mV. The passive properties of the cell (including membrane resistance and capacitance) were measured by hyperpolarizing the cell from − 70 to -80mV at the beginning of the step protocol [29, 30]. Transient inward current was calculated on the peak subtracting the baseline leak currents. Current density (pA/pF) was calculated as the ratio between the peak current recorded at + 20 mV and the capacitance of the cell. Inward current density was reported as a positive value [31, 32].

The holding potential was set according to the resting potential of the cells (between − 40 and − 80 mV). TTX 1µM was bath-perfused to abolish the recorded inward current thus confirming its identity. Additionally, QX-314 500 µM was dialyzed intracellularly to isolate the Na_v_-mediated current from other ionic currents, measured by mathematical subtraction of the residual current from the control. The organic cation N-methyl-D-glucamine (NMDG) was used to replace extracellular sodium in some experiments. Electrophysiological analysis and statistics were performed using Clampfit 10.6 (Molecular Devices) and OriginPro 9.1.

### Time lapse fluorescence imaging

To track the progression of the cell cycle in control condition and in the presence of TTX, cells were plated on black, Matrigel-coated 96-well plates that were then placed under an ImageXpress Micro Confocal system (Molecular Device). Pictures were taken at specific time intervals using a 20x Plan Apo objective, fractioning each well into 20 fields of view (FOVs). At each time point, cells were imaged for brightfield (48ms, 20% laser power), then FITC (105.26ms, 25% of 50% laser power, FL shading only) and finally TRITC (46.92ms, 50% of 50% laser power, FL shading only). Image acquisition was stopped after 56 h. Time-lapse analysis was performed within the Custom Module Editor of MetaXpress^®^ High-Content Image Acquisition and Analysis Software (“Custom Cell Cycle-20210928 – FUCCI”). A cell was considered as labeled when its average fluorescent amplitude was greater than two times the s.d. of the background noise. Non-labelled cells were selected automatically by the Analysis Software based on the morphological criteria and persistence in the field of view for at least 20 min. The experiment was replicated 3 times, each time with *n* = 6 wells for the control condition and *n* = 6 for 72 h-TTX pretreated wells. An average of 40 cells for each FOV was analyzed for a total of ~ 14,400 cells for condition.

### Western blot

Cell lysates were prepared either through the addition of a lysis buffer (LB) at 100 °C composed of 25mM Tris-HCl pH 6.8 (Sigma-Aldrich), 4% SDS (Euroclone), 20% Glycerol (Sigma-Aldrich) in deionized water, or through the addition of an ice-cold lysis buffer composed of 20mM Tris-HCl pH 8 (Sigma-Aldrich), 137mM NaCl (Sigma-Aldrich), 2mM EDTA (Sigma-Aldrich), 10% Glycerol (Sigma-Aldrich), and 1% NP-40 (Sigma-Aldrich), added with proteases and phosphatases inhibitor cocktail 1X (Thermo Fisher Scientific). Samples were sonicated for 15–20 min and centrifuged at 10,000 x g for 10 min; supernatants were collected, and the protein extracts were quantified using the Micro BCA™ Protein Assay Kit (Thermo Fisher Scientific).

Protein extracts (30 µg) were combined with NuPAGE LDS Sample buffer 4 × (0,5 M Tris-HCl, 40% Glycerol, 10% SDS, 0.04% Bromophenol Blue, Thermo Fisher Scientific) and heated at 95 °C for 3 min. Samples were then loaded onto an SDS-PAGE and run at constant voltage for 1–2 h. Separated proteins were transferred by Blotting System (Bio-Rad) to a nitrocellulose membrane (Amersham Protran, GE Healthcare) with 0.45 μm pore size and probed, in different combinations, with the following specific antibodies: rabbit 1:1000 anti-Vinculin (Sigma-Aldrich, Cat. No. V9131); rabbit 1:1000 anti-Tubulin (Cell Signaling, Cat. No. 2125 S); rabbit 1:1000 anti-SOX2, rabbit 1:1000 anti- C-MYC, rabbit 1:1000 anti-NANOG, rabbit 1:1000 anti-OCT4, rabbit 1:1000 anti-KLF4, rabbit 1:1000 anti-LIN28A (Cell Signaling, Cat. No. 9093); rabbit 1:1000 anti-MKi67 (Aviva Systems Biology, Cat. No. OOAN03278); rabbit 1:1000 anti-METRN (Aviva Systems Biology, Cat. No. OACD05531); rabbit 1:1000 anti-mTOR (Cell Signaling, Cat. No. 2983), rabbit 1:1000 anti-PHOSPHO-mTOR (Ser2448) (Cell Signaling, Cat. No. 2971); rabbit 1:1000 anti-PI3K p85a (ELK Biotechnology, Cat. No. EA206), rabbit 1:1000 anti-PHOSPHO p85-p55 PI3K (Y467/199) (ELK Biotechnology, Cat. No. ES6591); rabbit 1:1000 anti-p44-42 MAPK (ERK1/2) (Cell Signaling, Cat. No. 9102), rabbit 1:1000 anti-PHOSPHO p44-42 MAPK (T202/Y204) (Cell Signaling, Cat. No. 9101); mouse 1:1000 anti-GFAP (ValidAb™, Cat. No. HB8267); mouse 1:1000 anti-βIII Tubulin (ValidAb™, Cat. No. HB6639); mouse 1:4000 anti-GAPDH (ValidAb™, Cat. No. HB9177); rabbit 1:3000 anti-Calnexin (Genetex, Cat. No. GTX13504). Membranes were incubated O/N at 4 °C with primary antibodies, then 1 h with the secondary antibody, conjugated with HRP.

Membranes were then incubated with LiteAblot^®^TURBO (EuroClone) for 1 min in the dark and bands were detected using the ChemiDoc Touch^®^ imaging system (BioRad). The obtained bands were quantified by densitometry using Bio-Rad ImageLab 6.1 software.

### Immunofluorescence evaluations

#### Human GSCs

Control and treated cells were grown on coverslips (22 mm x 22 mm), placed in cell culture dishes (35 mm x 10 mm) until 90% confluence, fixed with 4% formalin (20 min), and post-fixed with 70% ethanol at -20 °C for at least 24 h. The samples were rehydrated for 10 min in PBS and then incubated with 1% Bovine Serum Albumin (BSA) to block nonspecific binding sites. Subsequently, the cells were immunolabeled using the following primary antibodies: mouse anti-Pan-Na_v_ sodium channel (1:500, Antibodies Incorporated, Cat. No. 75–405), the rabbit anti-pan-Na_v_ sodium channel (1:250, Sigma Aldrich, Cat. No. S6936), the human anti-E-Cadherin (1:200, Dako, Cat. No. M3612), the rabbit anti-SOX2 (1:700, Cell signaling, Cat. No. 3579), the rabbit anti-NANOG (1:100, Cell signaling, Cat. No. 3580), rabbit anti-MKi67 (1:250, Aviva Systems Biology, Cat. No. OOAN03278); the rabbit anti-Olig4 (1:200, Merk-Millipore, Cat. No. MAB345); the rabbit anti-METRN (1:100, Aviva Systems Biology, Cat. No. OACD05531) diluted in PBS (Sigma-Aldrich) for 1 h at RT in a dark, moist chamber. Then, the cells were washed with PBS and incubated 45 min with goat anti-mouse IgG (H + L) highly cross-adsorbed secondary antibody, Alexa Fluor Plus 594, mouse anti-human IgG (H + L) highly cross-adsorbed secondary antibody, Alexa Fluor Plus 594 and goat anti-rabbit IgG (H + L) highly cross-adsorbed secondary antibody, Alexa Fluor Plus 488 diluted 1:200 in PBS (Thermo Fisher Scientific). DNA counterstaining was performed using 0.1 mg/mL Hoechst 33,258 (Sigma-Aldrich). Lastly, cells were mounted with a drop of Mowiol (Calbiochem-Inalco) for microscopy visualization. Sections were examined using a Leica DM6B WF microscope (Leica microsystems), images were captured with an ORCA-Flash4.0 V3 Digital CMOS camera C13440-20CU (Hamamatsu Photonics), and ImageJ software (Version 1.51) was used to measure the mean optical density (OD) (ratio of the mean of immunofluorescence intensity on the cell surface). To prevent potential discrepancies in results caused by slight procedural variations, all immunostaining reactions were simultaneously performed and, as a control, some sections were incubated without primary antibodies, using only PBS; any immunoreactivity was observed under this condition.

For each condition, 11 quadrants (about 50 cells) were evaluated for random analysis. Single channel images were analyzed in grayscale, where the minimum value was 0 (black) and the maximum value was 255 (white).

Alternatively, spheroids were fixed in 1% *paraformaldeide* (PFA) for 30 min at RT, washed 2X with PBS and stored at 4 °C in PBS. Then, spheroids were gently manipulated with a cut sterile p1000 pipette tip and cryoprotected O/N in 30% sucrose/PBS at 4 °C. The following day, organoids were embedded in OCT compound (Bio-Optica) and snapped frozen in dry ice. 20 μm sections were serially cut using a cryostat and collected on Superfrost plus slides for immunofluorescence analysis. Samples were washed in PBS for 5 min and blocked in 1% BSA for 1 h, at RT, followed by overnight incubation using abovementioned primary antibodies. Then, organoids were washed in PBS for 5 min each and incubated for 1 h with secondary antibodies. Nuclei were stained with 0.1 µg/mL Hoechst 33,258 (Sigma Aldrich). After washing with PBS for 5 min, coverslips were mounted with Mowiol (Calbiochem). The images acquisition and analysis were performed as abovementioned.

#### GBM biopsies imaging

Sections of human GBM biopsies already fixed in PFA 4%, were kindly provided by the IOV (Istituto Oncologico Veneto, Padova, IT). Immunostaining was performed to detect the expression of Na_v_ channels and GSC stemness markers in these biopsies. The stained sections were then imaged using a confocal microscope. Images of the immunostained human GBM biopsies were acquired using the TCS SP8 X confocal microscopy system (Leica, Wetzlar, Germany) at the Centro Grandi Strumenti (CGS) of the University of Pavia. The imaging was conducted with the following settings: for Na_v_-Pan, the excitation peak was at 480 nm and the emission at 500–550 nm; for GSC stemness markers, the excitation peak was at 647 nm and the emission at 660–780 nm. A 40X oil immersion objective (NA = 1.3) was used for imaging. The following primary antibodies were used for immunostaining: mouse anti-pan-Nav sodium channel (1:250, Sigma Aldrich, Cat. No. S6936), rabbit anti-SOX2 (1:700, Cell Signaling, Cat. No. 3579), and rabbit anti-CD133 (1:200, Cell Signaling, Cat. No. 60577). GBM biopsy sections were incubated with primary antibodies against pan-Nav sodium channel, SOX2, and CD133 at the specified dilutions. Nuclei were.

stained with 0.1 µg/ml Hoechst 33,258 (Sigma–Aldrich). Following primary antibody incubation, appropriate Alexa Fluor 488 and 647 secondary antibodies conjugated with fluorescent dyes (1:200, Thermo Fisher Scientific) were applied. Confocal images were acquired with the specified settings for excitation and emission peaks using the TCS SP8 X confocal system.

*We have included below a table with the clinical information for human tumor tissues (*Table 2*).*

**Table 2:** Clinical annotations for the human tumor tissues investigated

Code	Sex	Age at diagnosis	Location	Primary/ Recurr.	WHO grade	Molecular Subtype	IDH and 1p/19q co-del. status	Ki67 (%)	MGMT	IDH	Dead/ Alive	Overall survival (months)
1	M	58	Fr. lobe L	*P*	IV	N/A	N/A	N/A	37%	WT	Dead	15
2	M	47	Occ. lobe L	*P*	IV	N/A	N/A	N/A	Met	WT	Alive	23
3	M	47	Encephalon	*P*	IV	N/A	N/A	N/A	Unmet	WT	Dead	28
4	F	48	Fr. lobe L	*P*	IV	N/A	N/A	N/A	55%	WT	Alive	33

### Establishment of the mouse allograft model of TMZ sensitivity

Glioma induction: Adult C57BL/6J mice were housed under a 12-hour light/dark cycle with food and water available ad libitum. All experimental procedures adhered to the relevant guidelines and were approved by the Italian Ministry of Health (protocol number 981/2020PR). Animals were randomly allocated to experimental groups. GL261 cells were cultured as described above. One experimental mice group was stereotactically injected with 40,000 GL261 cells (20,000 cells/µl PBS solution) that had been treated for 72 h with Tetrodotoxin (TTX, HelloBio, 30 µM in DMEM), while another group was injected with untreated GL261 cells. Injections were targeted to the forelimb motor cortex (coordinates from bregma: 1.75 mm lateral to the midline, 0.5 mm anterior [33] using a Hamilton syringe, and an automated pump (KdScientific, US) at a depth of 0.9 mm from the pial surface.

Temozolomide administration: Following the in vivo injection of GL261 cells, TMZ treatment began the day after cell injection. TMZ was dissolved in a solution of 25% of Dimethyl Sulfoxide (DMSO, Sigma Aldrich, USA) made in saline. TMZ was administered via a single daily intraperitoneal (i.p.) injection for tumor treatment to both experimental groups. For the intraperitoneal injections, a stock TMZ solution (4 mg/ml in 25% DMSO/saline) was prepared. The effective dose was 40 µg of TMZ per gram of body weight, corresponding to 10 µl of TMZ stock per gram of animal weight. Mice were sacrificed 2 weeks after tumor induction [34, 35].

Immunohistochemical analyses: Two weeks following tumor induction, mice were transcardially perfused with phosphate-buffered saline (PBS, Sigma Aldrich, USA) followed by a fixative solution containing 4% paraformaldehyde in 0.1 M sodium phosphate buffer (pH 7.4). The brains were then dissected, post-fixed for 2 h at 4 °C, cryoprotected in 30% sucrose, and subsequently frozen. Coronal sections of 50 μm thickness were obtained using a sliding microtome (Leica, Germany). The sections were stained with Hoechst dye (#B2883; 1:500; Bisbenzimide, Sigma Aldrich, USA) for nuclei visualization and tumor size quantification, and with Ki67 (Abcam 16667; 1:400) to indicate proliferating cells, following antigen retrieval with citrate buffer (pH 6, 10 mM) for 30 min at 80 °C. Tumor volume for each animal was calculated by summing all damaged areas and multiplying by the section thickness and the spacing factor. Fluorescent images were acquired using a Zeiss Axio Observer microscope equipped with a Zeiss AxioCam MRm camera (Carl Zeiss MicroImaging GmbH, Germany). Images for each slice were captured with a 10x objective and stitched into a single composite image using Zen Blue Edition software (Carl Zeiss MicroImaging GmbH, Germany). For the evaluation of the proliferation index, high-magnification single z-stack images were acquired using a Zeiss Airyscan Confocal Microscope (Carl Zeiss MicroImaging GmbH, Germany) equipped with a 40× oil objective. Cells labeled with Ki67 and Hoechst were counted using the Cell Counter (ImageJ software). The proliferation index was calculated as the proportion of Ki67-positive cells relative to the total number of Hoechst-labeled cells. This analysis was performed in three distinct fields of view for each of the three coronal slices representing the central region of the brain tumor. Typically, GL261-injected cells occupy a different z-plane compared to brain neurons.

Behavioral tests: The body weight of the mice was monitored daily to detect weight loss during tumor development. Three different behavioral tests were administered: the grip strength test to evaluate forelimb strength, the grid walk test to assess motor behavior [35], and the tail suspension test to measure depressive-like states. The experimental protocol for characterizing the motor glioblastoma model included a baseline assessment (day 0) and subsequent evaluations on days 3, 6, 10, and 13. To evaluate changes in behavior following tumor induction, the results of each test on the various days were normalized to the baseline performance. In the grip strength test, a peak amplifier automatically measured the peak pull-force achieved by the animals’ forelimbs [36]. The cage mobility test was performed for 5 min (or for grid walk task, mice were placed on a wire mesh for 5 min), and the total distance traveled (in centimeters) was measured using a personally developed computerized system for background subtraction, centroid identification, and tracking. Additionally, the tail suspension test was conducted for 6 min. Immobility time was measured during the final 4 min, as nearly all mice attempt to escape during the first 2 min. The total amount of immobility time, defined as the period during which the animal is hanging passively and motionless, was measured for each animal and considered an index of “depression-like” behavior [37].

### Statistical analysis

All data are expressed as mean ± SEM. No data was excluded from analysis. To confirm a normal distribution of the dataset, a QQ plot was generated, and the Shapiro-Wilk test was performed for each pool of data. If a normal distribution was not confirmed, a nonparametric test was performed. Wilcoxon rank-sum test was used to compare two independent samples for non-normal distributions; Wilcoxon signed-rank test was used to conduct a paired difference test of repeated measurements on a single sample to assess whether their population mean ranks differ. Mann-Whitney Test was applied to compare significance between two sample means that come from the same population. Repeated-measures ANOVA, between-subjects factors ANOVA, mixed-factors ANOVA, were used to test for statistical differences between multiple experimental conditions. Tukey’s multiple comparison test was used to determine which means amongst a set of means differ from the rest. Statistical analyses were performed using Prism (GraphPad Software Inc) or Origin 2021 (OriginLab) and considered significant if *p* < 0.05. Power analyses were performed using G*power.

## Results

### Na_v_ upregulation is observed in TMZ-resistant GSCs and it is associated with reduced survival and increased expression of stemness markers expression in GBM patients

**Fig. 1:**
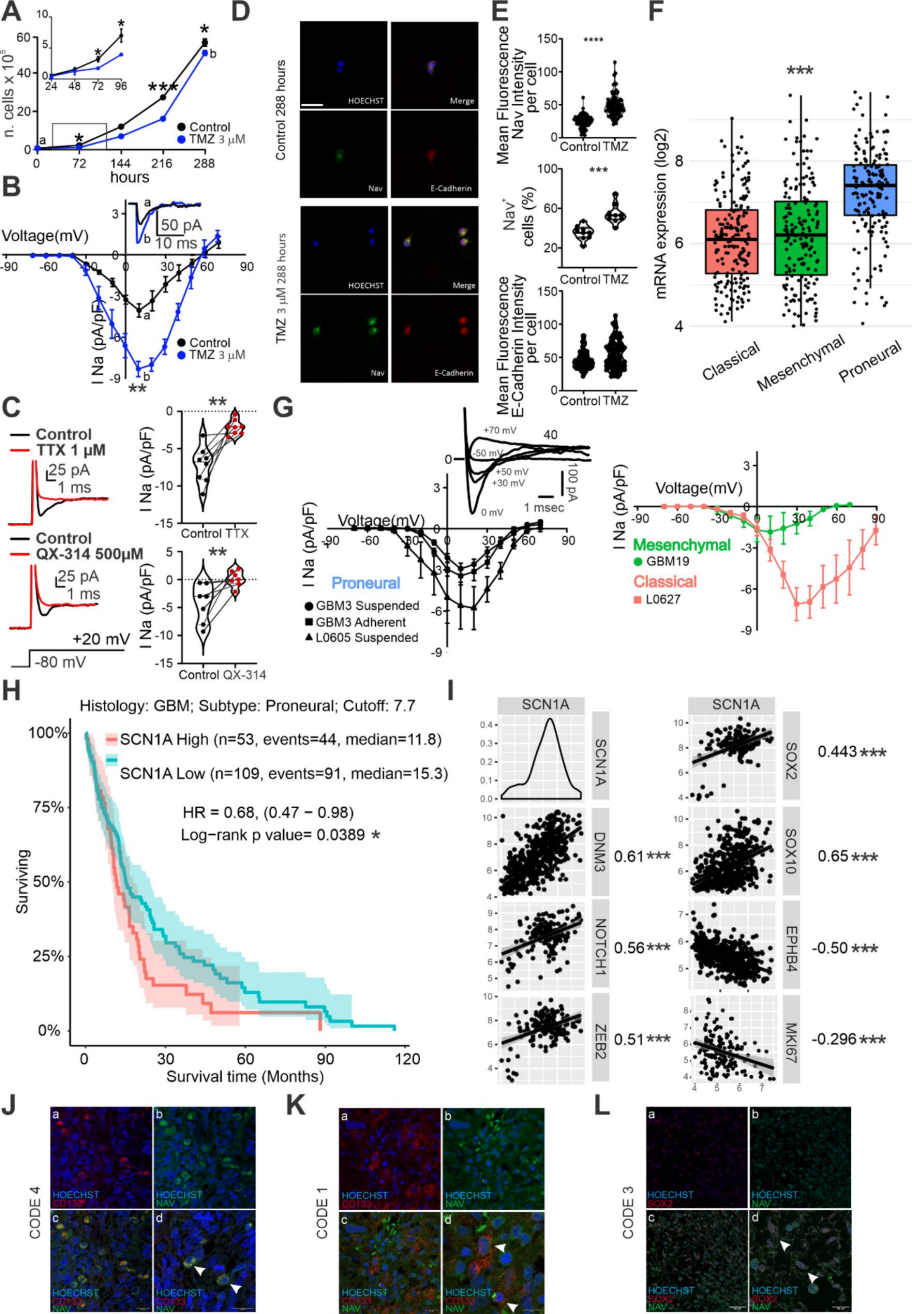
Expression of SCN1A is associated with worse survival in glioblastoma patients and correlates with GSCs markers: (**A**) Pooled data for the 288-hours proliferation assays of proneural GSCs in the control condition (circles) and in the presence of TMZ 3µM (squares). Inset: the same pool of data but for the time window zoomed in the interval from 0 to 96 h. (**B**) Average I-V Plot from − 70 mV to 70 mV acquired in whole-cell patch clamp showing a significant inward current upregulation in TMZ-resistant cells (b, TMZ 3µM) compared to non-treated cells (a, Control). Inset: representative inward current traces recorded at + 10 mV for the two conditions. (**C**) Sensitivity to TTX and QX-314 revealed the nature of the recorded inward current. On top, the Na_v_-mediated nature of the current was assessed with bath perfusion of the specific blocker Tetrodotoxin (TTX 1µM). Representative trace in control (black) and after TTX perfusion (red). On the bottom, the Na_v_ -mediated current was also tested with intracellular dialysis of the lidocaine derivative QX-314 500 µM, which blocks the channel from the cytosolic compartment. Representative traces in control (black) and after QX-314 dialysis (red) are also displayed. After both treatments, the inward current was significantly abolished, thus confirming the current’s identity. On the right, average pool data for all the recorded cells in control, with TTX and QX-314 perfusion. (**D**) Immunoreactivity to the Pan-Na_v_ antibody (green channel), E-Cadherin antibody (red channel) and nuclear staining (Hoechst, blue channel) in control condition (CTR) and after 288 h TMZ 3 µM treatment. Scale bar is 50 μm. (**E**) Optical density (OD) violin plots illustrating single-cell measurements in CTR condition and after 288 h TMZ 3 µM treatment (upper panel). Pool data for the percentage of cells positive to Na_v_ for the two conditions (lower panel).(**F**) Bulk analysis of SCN1A mRNA expression levels in three different GBM subtypes. (**G**) Whole-cell electrophysiological recordings were performed on different subtypes of GSCs, and the Na_v_-mediated current was recorded. On the top, representative traces are recorded at different holding potentials from a proneural line. The corresponding voltage steps applied are displayed. On the bottom, the average I-V plot from − 70 mV to 70 mV for all the proneural recorded primary cell lines. (left) Recordings of adherent and suspended proneural GSCs. (right) Recordings of the classical and mesenchymal cell line. (**H**) Kaplan-Meier survival curve of GBM patients with a proneural subtype. The cut-off was set at 7.7 (log2). (**I**) SCN1A mRNA expression level positively correlates with some stemness markers. The slope value of the linear fitting and the significance of the correlation are displayed. Conversely, SCN1A negatively correlates with markers associated with proliferation and differentiation. (**J**-**L**) Immunohistochemical staining for different GSC markers (CD133, SOX2) and Nav in various GBM patient biopsies. Panels a and b show representative images of staining for Hoechst and the GSC marker, and Hoechst and Nav, respectively. Panel c shows the merge of the previous two panels. Scale bar is 20 μm. Panel d shows a more defined FOV taken from panel c. Scale bar is 20 μm. The white arrows indicate cells positive for both Nav and the GSC markers

TMZ constitutes the standard therapy for GBM, but in most patients a relapse occurs after prolonged administration due to gained resistance to the treatment [38]. To model GBM tumor relapse in vitro, we maintained cultured GSCs [39] under a suboptimal concentration (3µM, Supplementary Fig. 1A) of the chemotherapeutic TMZ for up to 12 days (288 h). We measured cell growth in terms of number of cells (Fig. 1A), in control condition (blacks) and in the presence of TMZ 3µM (blue). As expected, the total cell number was significantly reduced (*p* = 0.015, Two-way ANOVA) from 72 h (Fig. 1A **insert**) to 216 h in the presence of TMZ (72 h control: 2.36 ± 0.09 × 10^5^ cells; 72 h TMZ: 1.20 ± 0.14 × 10^5^ cells; *n* = 4; *p* = 0.011, Tukey’s multiple comparisons test; 216 h control: 27.30 ± 0.30 × 10^5^ cells; 216 h TMZ: 16.16 ± 0.72 × 10^5^ cells; *n* = 4; *p* < 0.0001, Tukey’s multiple comparisons test), suggesting that TMZ treatment effectively impacted cell proliferation *in vitro.* However, GSCs resumed an exponential growth after 216 h (288 h control: 55.81 ± 1.89 × 10^5^ cells; 288 h TMZ: 50.38 ± 1.33 × 10^5^ cells; *n* = 4; *p* = 0.031, Tukey’s multiple comparisons test; Fig. 1A), showing reduced sensitivity to the chemotherapy. Consistently, the number of dead cells was higher in TMZ condition until 216 h but decreased at 288 h, compared to control (Supplementary Fig. 1B).

To investigate the cellular mechanism of chemotherapy resistance, we compared the repertoire of membrane channel conductances before and after TMZ treatment using whole-cell patch clamp (Fig. 1B). GSCs responded to prolonged TMZ exposure with a robust upregulation of an inward current, reaching its peak upon depolarization to + 10 mV, from a holding potential of -70 mV (control: 4.1 ± 1.1 pA/pF, *n* = 12, black trace; TMZ: 8.8 ± 0.6 pA/pF, *n* = 10, blue trace; *p* = 0.0051, Mann-Whitney test). This effect was not dependent on the time spent by the GSCs in the medium (Supplementary Fig. 1C). This inward current, both in control and in TMZ-resistant GSCs, had a reversal potential around + 55 mV and was abolished by Tetrodotoxin (TTX, 1 µM) bath perfusion (control: 7.2 ± 2.4 pA/pF, *n* = 8, black trace; TTX: 2.1 ± 0.9 pA/pF, *n* = 8, red trace; *p* = 0.0078, Wilcoxon test; Fig. 1C top) and by QX-314 (500 µM) intracellular dialysis (control: 3.4 ± 1.3 pA/pF, *n* = 7, black trace; QX-314: 0.81 ± 0.91 pA/pF, *n* = 7, red trace; *p* = 0.0156, Wilcoxon test; Fig. 1C bottom). For this reason, we classified it as voltage-gated sodium channel (Na_v_)-mediated inward current. To clarify the upregulation of this functional Na_v_ current density, we evaluated the sodium channel protein subunit alpha (SCN1) expression in control condition and after 288 h TMZ 3 µM treatment, by performing a co-immunostaining with E-cadherin as a major plasma membrane protein (Fig. 1D and Supplementary Fig. 1D). Consistently with Na_v_ current upregulation, we observed a significant increase both in the Optical Density (OD) and in the percentage of Na_v_ positive cells upon 288 h TMZ treatment (OD control: 24.90 ± 1.00, *n* = 90 cells; OD TMZ: 49.01 ± 1.77, *n* = 90 cells; *p* < 1 × 10^− 14^ Mann-Whitney test; Fig. 1E, upper panel; percentage of Na_v_ positive cells control: 35.32 ± 2.47, *n* = 9 cells; percentage of Na_v_ positive cells under TMZ: 54.33 ± 3.37, *n* = 9 cells; *p* = 0.00033 Mann-Whitney test; Fig. 1E, middle panel) while E-cadherin OD remained unchanged between the two conditions (OD control: 45.62 ± 1.35, *n* = 90 cells; OD TMZ: 54.49 ± 2.21, *n* = 90 cells; *p* = 0.08 Mann-Whitney test; Fig. 1E, lower panel).

Na_v_ is predominantly expressed in excitable cells such as neurons [40] and cardiac cells [41]. However, its presence is minimal in glial cells [42], and little is known about its role in GBM [43] (Supplementary Fig. 1E). We exploited GlioVis repository (TCGA_GBM) [44] to investigate the mRNA expression of SCN1A in GBM human samples (Fig. 1F) as one of the most representative Na_v_ transcript isoforms in GBM (Supplementary Fig. 1F). Overall, SCN1A mRNA expression level was significantly higher in non-tumoral samples compared to GBM tissue (non-tumor: 7.7 ± 0.2 log2, n = 10; GBM: 6.5 ± 1.2 log2, n = 538; *p* = 1.9E-03, Bonferroni correction). However, a group of GBM samples presented SCN1A expression level similar to non-tumoral tissue and significantly different from the rest of the GBM sample, and it was mainly identified as the proneural GBM subtypes [45] (Proneural: 7.4 ± 1.1 log2, n = 163; Mesenchymal: 6.2 ± 1.2 log2, n = 166; Classical: 6.1 ± 1.1 log2, n = 199; *p* = 4.6E-19 Proneuronal vs Classical, Bonferroni correction; *p* = 3.9E-15 Proneuronal vs Mesenchymal, Bonferroni correction, Fig. 1F). To determine whether the differential abundance of Na_v_ transcripts, based on GBM subtypes, correlated with distinct functional expression of the channel, whole-cell patch clamp experiments were conducted on GSCs, separated according to their proneural, mesenchymal, and classical subtypes (Fig. 1G). Consistently with the bulk mRNA analysis reported in TCGA_GBM repository, proneural GSCs exhibited a significantly higher functional current density (Proneural “GBM3” cells growth in suspension: 2.9 ± 0.4 pA/pF, n = 7/17; Proneural “GBM3’’ adherent cells: 3.5 ± 0.7 pA/pF, n = 5/14; Proneural “L0605” in suspension: 5.3 ± 1.4 pA/pF, n = 8/18; Mesenchymal (GBM19): 1.8 ± 1.1 pA/pF, n = 5/16; *p* = 0.002 One-Way Anova; Fig. 1G). Conversely, GSCs identified with a classical profile (L0627) exhibited an inward current (Classical “L0627” in suspension: 6.9 ± 1.2 pA/pF, n = 6/7) with a peak shifted towards more depolarized potentials (of about 30 mV) and a reversing value around + 110 mV, consistent with a calcium-mediated conductance. When analyzing all GBM patients with an identified proneural subtype in a pooled setting and selecting the 7.7 log2 value as the cut-off point for SCN1A transcript expression, the 120-month overall survival (OS) rate, as depicted in the Kaplan-Meier survival curves (Fig. 1H), was significantly lower (*p* = 0.039; log-rank test) ) for those with SCN1A transcript levels higher than 7.7 log2 (red curve) compared to those with expression levels below the cut-off (green curve). Collectively, Kaplan-Meier analyses revealed a significantly reduced survival rate for proneural GBM patients with elevated SCN1A expression. Furthermore, we correlated SCN1A expression levels with all available mRNA quantifications in GlioVis (Fig. 1I), revealing a positive correlation between SCN1A expression and mRNA transcripts generally associated with stemness (among which SOX2 and SOX10), while showing a negative correlation with transcripts linked to proliferation or differentiation (EPHB4 and MKI67).

To validate the expression of Na_v_ in human GBM, we analyzed biopsies from 4 patients (GBM information is listed in the Methods section). In 3 out of 4 patients, positivity for the Na_v_-Pan antibody was detected, and in some cells, we observed dual positivity for Na_v_ and GSC markers such as CD133 and SOX2. Panels a and b (Fig. 1J-L) show representative images of staining for Hoechst and the GSC marker, and Hoechst and Na_v_, respectively. Panel c shows the merge of the previous two panels, and panel d shows more defined FOV taken from c. The white arrows indicate cells positive for both Na_v_ and the GSC markers, supporting a potential association between Na_v_ expression and GSC characteristics in GBM biopsies.

Overall, the data presented in Fig. 1 suggest a positive correlation between Na_v_ overexpression and chemotherapy resistance in GSCs, which is strongly associated with a worse outcome in GBM patients with a proneural subtype. Additionally, the presence of Na_v_ in human GBM biopsies, particularly in cells also positive for GSC markers, suggest a possible relevance of Na_v_ expression in maintaining stem-like characteristics in GBM.

### Blocking Na_v_ significantly reduces the expression of stemness markers in GSCs and their self-renewal ability

**Fig. 2:**
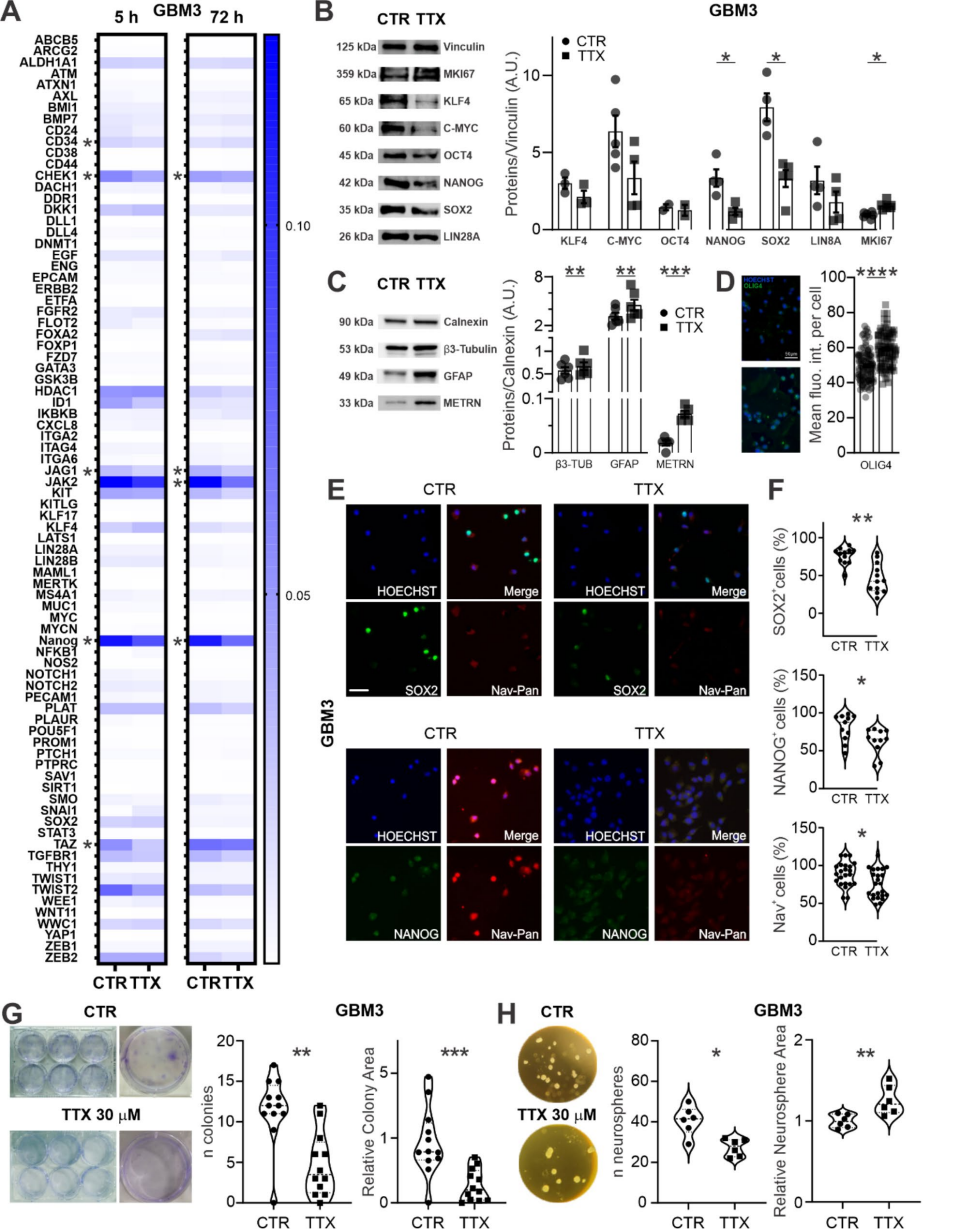
GSCs stemness markers as well as self-renewal properties are significantly downregulated when Na_**v**_ is pharmacologically blocked: (**A**) Heatmap showing the differential expression level of the mRNA stemness markers (gradient bar represents the degree of gene expression level) in the control condition and after 5 h and 72 h of TTX 30 µM treatment in the medium. The measurement is reported in Log2FoldChange. The degree of significance is specified on the left side of the marker name. (**B**) WB quantification for stemness markers reveals a significant reduction in the protein content for SOX2 and NANOG. (**C**) Western Blot quantification for differentiation markers (**E**). (**D**) Immunoreactivity to O4 antibody in control and after 72 h of TTX treatment. (**E**) Top: immunoreactivity to Pan-Na_v_ antibody (red channel), SOX2 (green channel), and nuclear staining (Hoechst, blue channel) for GSCs in the control and after 72 h of TTX treatment. Scale bar is 50 μm. Bottom: Immunoreactivity to the Pan-Na_v_ antibody (red channel) and positivity for NANOG (green channel) and nuclear staining (Hoechst, blue channel) for GSCs in the control and after 72 h of TTX treatment. Scale bar is 50 μm.(**F**) Pool data for the percentage of cells positive to SOX2, NANOG and Nav for the two conditions. (**G**) Representative example of a clonogenic assay in the control (top) and with cells pretreated for 72 h with TTX 30 µM (bottom). Pool data quantifying the number and the relative area of the colonies (on the right). (**H**) Representative example of a neurosphere formation assay in the control (top) and with cells pretreated for 72 h with TTX (30 µM) (bottom). Pool data quantifying the number and the relative area of the neurospheres

To explore the functional relationship between Nav expression and stemness regulation in GBM, GSCs (GBM3) were conditioned with the Na_v_ blocker tetrodotoxin (TTX, 30 µM) for both 5 h and 72 h. Subsequently, mRNA expression for 80 markers associated with cancer stem cells was quantified (Fig. 2A), revealing a general decrease in stemness markers expression at both time points. After 5 h of TTX treatment, significant reductions were observed in the following markers: CD34 (control: 0.017 ± 0.002 log2 fold change, *n* = 3; TTX: 0.013 ± 0.001 log2 fold change, *n* = 3; *p* = 0.015 Two-way ANOVA), CHEK1 (control: 0.060 ± 0.004 log2 fold change, *n* = 3; TTX: 0.043 ± 0.003 log2 fold change, *n* = 3; *p* = 0.008 Two-way ANOVA), JAG1 (control: 0.032 ± 0.003 log2 fold change, *n* = 3; TTX: 0.022 ± 0.002 log2 fold change, *n* = 3; *p* = 0.022 Two-way ANOVA), NANOG (control: 0.147 ± 0.009 log2 fold change, *n* = 3; TTX: 0.097 ± 0.007 log2 fold change, *n* = 3; *p* = 0.004 Two-way ANOVA), and TAZ (control: 0.055 ± 0.004 log2 fold change, *n* = 3; TTX: 0.026 ± 0.002 log2 fold change, *n* = 3; *p* = 0.012 Two-way ANOVA). As for the 72 h treatment, this included significant differences for CHECK1 (control: 0.086 ± 0.032 log2 fold change, *n* = 4; TTX: 0.002 ± 0.012 log2 fold change, *n* = 4; *p* < 0.0001 Two-way ANOVA), JAG1 (control: 0.040 ± 0.002 log2 fold change, *n* = 4; TTX: 0.013 ± 0.006 log2 fold change, *n* = 4; *p* = 0.032 Two-way ANOVA), JAK2 (control: 0.116 ± 0.011 log2 fold change, *n* = 4; TTX: 0.047 ± 0.025 log2 fold change, *n* = 4; *p* < 0.0001 Two-way ANOVA), NANOG (control: 0.138 ± 0.022 log2 fold change, *n* = 4; TTX: 0.047 ± 0.025 log2 fold change, *n* = 4; *p* < 0.0001 Two-way ANOVA). Western blot analysis of the pluripotency transcription family also demonstrated a significant downregulation of stemness-related proteins, including NANOG and SOX2 (NANOG control: 3.3 ± 0.3 a.u., *n* = 4; NANOG TTX: 1.2 ± 0.2 a.u., *n* = 4; *p* = 0.04 Multiple paired t-test; SOX2 control: 7.9 ± 0.9 a.u., *n* = 4; SOX2 TTX: 3.3 ± 0.7 a.u., *n* = 4; *p* = 0.01 Multiple paired t-test; Fig. 2B, Supplementary Fig. 2A, B). Conversely, MKI67, a cellular marker for proliferation, was upregulated (MKI67 control: 0.9 ± 0.1 a.u., *n* = 6; MKI67 TTX: 1.6 ± 0.1 a.u., *n* = 6, *p* = 0.005 Multiple paired t-test, Fig. 2B) as well as Meteorin (METRN), a protein involved in glial cell differentiation (METRN control: 0.018 ± 0.005 a.u., *n* = 5; METRN TTX: 0.072 ± 0.006 a.u., *n* = 6, *p* = 0.0008 Multiple paired t-test, Fig. 2C). To further investigate the impact of Nav channel blockade on GSC differentiation, we also performed Western Blot analysis for the following markers: GFAP (a marker for astrocytes) and β-tubulin III (a marker for neurons) (GFAP control: 3.15 ± 0.41 a.u., *n* = 6; GFAP TTX: 4.50 ± 0.67 a.u., *n* = 6; *p* = 0.0085 Multiple paired t-test; β-tubulin III control: 0.57 ± 0.07 a.u., *n* = 6; β-tubulin III TTX: 0.67 ± 0.08 a.u., *n* = 6; *p* = 0.0087 Multiple paired t-test; Fig. 2C). Additionally, immunostaining for the oligodendrocyte marker O4 (OLIG4) was conducted since the available antibody was not compatible for Western Blot application (OLIG4 control: 49.8 ± 0.9 mean fluorescent intensity per cell, *n* = 100: OLIG4 TTX: 59.1 ± 0.9 mean fluorescent intensity per cell, *n* = 100, *p* < 0.00001, Mann-Whitney test, Fig. 2D).

To validate that the decrease in SOX2 and NANOG protein levels correlated with a reduction in the percentage of cells positive to these markers, we conducted co-immunostaining for Hoechst, SOX2, and Na_v_ in GSCs following a 72 h treatment with TTX 30 µM. In comparison to the control condition, we observed a significant decrease in the number of cells positive for SOX2 upon TTX treatment (control: 76.0 ± 3.2%, *n* = 12 wells; TTX: 46.3 ± 5.7%, *n* = 12 wells; *p* = 0.00015 Mann-Whitney test; Fig. 2E, F and Supplementary Fig. 2D). Interestingly, a significant co-expression between SOX2 and Na_v_-Pan positivity was also noted (Supplementary Fig. 2E). We then assessed the change in the fraction of cells positive for NANOG. Consistently, we identified a significant decrease in the number of cells positive for NANOG upon TTX treatment (control: 79.9 ± 3.2%, *n* = 11 wells; TTX: 61.8 ± 5.1%, *n* = 11 wells; *p* = 0.029 Mann-Whitney test; Fig. 2E, F). Moreover, a notable reduction was observed in the percentage of cells positive for Na_v_ (control: 67.6 ± 4.8%, *n* = 23 wells; TTX: 47.8 ± 5.2%, *n* = 23 wells; *p* = 0.018 Mann-Whitney test; Fig. 2E, F, Supplementary Fig. 2D-G).

As GSCs are characterized by their self-renewal capability [25], we hypothesized that the pharmacological blockade of Na_v_ should lead to a decrease in the self-renewal properties of GSCs. To assess this, clonogenic and neurosphere assays were performed, as previously reported [46] (see also Materials and Methods). Following the standard 72 h treatment with TTX 30 µM, a significant decrease in both colony formation (control: 11.8 ± 4.1, *n* = 12; TTX: 4.1 ± 0.8, *n* = 18; *p* < 0.0001 Mann-Whitney test; Fig. 2G) and colony area (control: 1.0 ± 0.5 normalized area, *n* = 11; TTX: 0.4 ± 0.1, *n* = 17; *p* = 0.0003 Mann-Whitney test; Fig. 2G) was observed in GSCs compared to the control condition. Moreover, when evaluating the effect of TTX treatment on neurosphere formation, a significant decrease in the number of neurospheres (control: 40.7 ± 7.2, *n* = 6 number of neurospheres; TTX: 27.3 ± 1.7, *n* = 6 number of neurospheres; *p* = 0.0152 Mann-Whitney test; Fig. 2H) was reported, along with a significant increase in their area (control: 0.99 ± 4.1, *n* = 12 normalized area; TTX: 1.25 ± 0.07, *n* = 6 normalized area; *p* = 0.0087 Mann-Whitney test; Fig. 2H).

Taken together, our data support the hypothesis that Na_v_ functional expression is linked to stemness, and its pharmacological blockade decreases the self-renewal properties of GSCs while promoting proliferation and differentiation.

### The functional expression of Na_v_ channels is markedly diminished in differentiated cells or under conditions of extracellular acidification

To verify the correlation between the functional expression of Na_v_ channels and the stemness profile of GSCs, we added Retinoic Acid (RA, 10 µM) to the medium of GBM3 cells for 5 days or, alternatively, 10% Fetal Bovine Serum (FBS) for 6 days (Supplementary Fig. 3). Both RA and FBS possess the ability to induce cell differentiation [47–49], and both treatments led to changes in the electrophysiological characteristics of the recorded cells, in the amount of cells exhibiting a Na_v_-mediated current and in the current density of the latter (Supplementary Fig. 3A-F). Consistent with the downregulation of Nav currents, we observed a significant decrease in Optical Density (OD) when comparing the control condition to cells treated with RA 10 µM or 10% FBS (Supplementary Fig. 3G, H). Downregulating the functional Nav current resulted in no difference in sensitivity to TMZ, regardless of whether GSCs were pre-treated with TTX or not (Supplementary Fig. 3I, J). These changes were also associated with an increased in the potassium inward current and a significant drop in the resting membrane potential (Supplementary Fig. 3K-M).

In light of recent reports suggesting the influence of pH on Na_v_ channel modulation [50], we examined the effect of pH on GSCs. Cells incubated at pH 6.8 for 24 h exhibited a pronounced downregulation of Na_v_ compared to the control condition at pH 7.2 (Supplementary Fig. 3N-P), demonstrating a pH-dependent modulation of the channel.

### Na_v_ is functionally expressed in a cell-cycle specific manner and is necessary to maintain GSCs in the G2/M and G0 phase through the regulation of the resting membrane potential

**Fig. 3:**
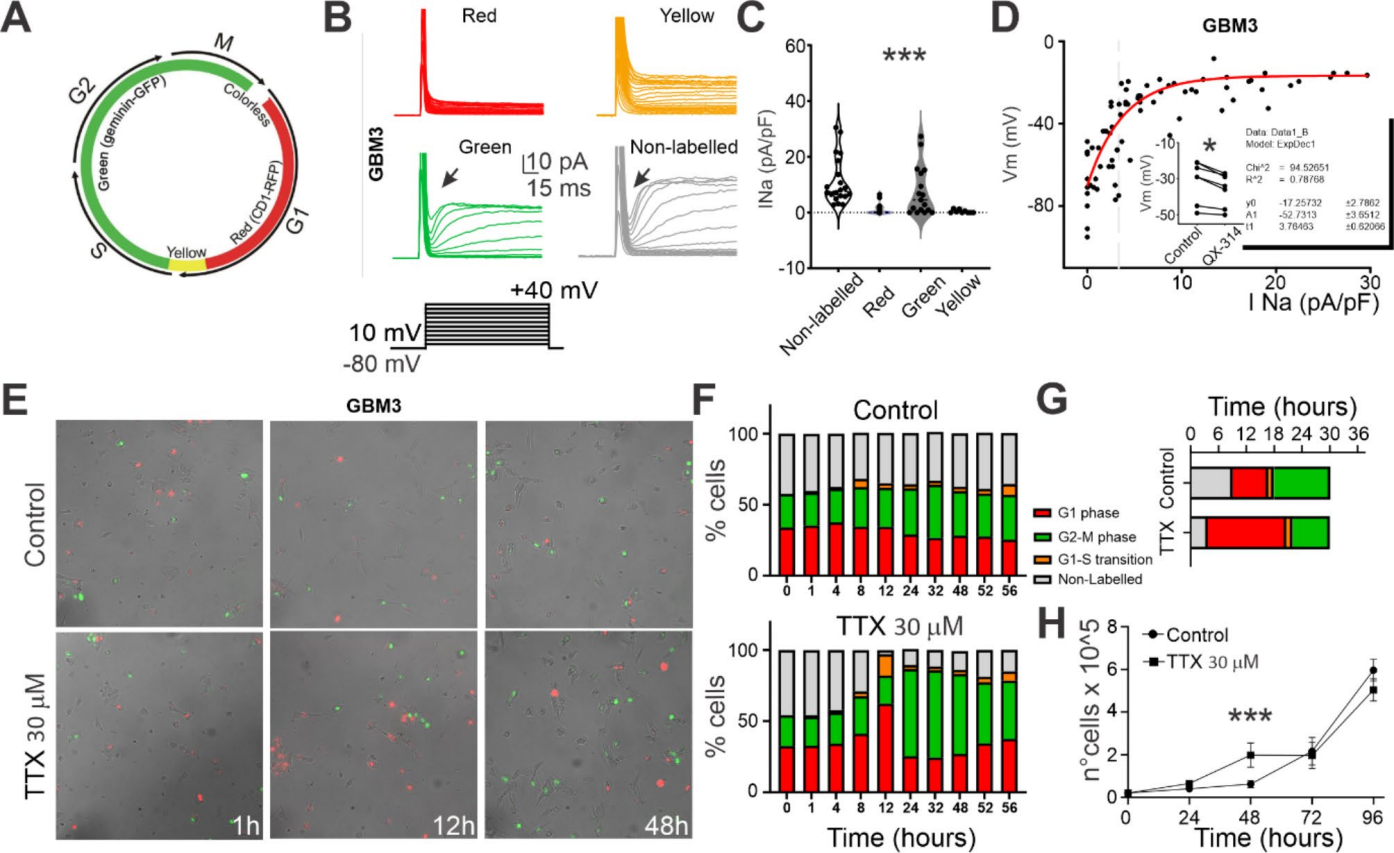
Na_v_ is functionally expressed in a cell-cycle phase-specific manner and regulates the G0 to G1 transition: (**A**) Cell cycle progression was investigated by viral-mediated expression of the Fluorescence Ubiquitin Cell Cycle Indicator (FUCCI) system. A scheme of the FUCCI system is provided. (**B**) Representative electrophysiological traces from primary GSCs in different cell cycle phases recognized by fluorescence positivity. (**C**) Pool data for all the recorded cells in different cell cycle phases revealed a significantly higher functional expression of Na_v_ in GSCs in the G0 (Non-labeled) and G2/M phases (green). (**D**) Correlation between Na_v_ current density and resting membrane potential for each recorded cell. Inset: change in the RMP as a consequence of QX-314 intracellular dialysis (5 min after break-in). (**E**) Time-lapse example images acquired at 1 h, 12 h, and 48 h for the 56 h time-lapse recording experiment. (**F**) Average distribution of GSCs in cell cycle phases: the mean percentage distribution of green cells, red cells, or green and red has been quantified in control and after the application of TTX. (**G**) Cell cycle phases average duration for a pool of representative GSCs tracked for the whole time lapse (30 h) in control condition and in the presence of TTX (**H**) 96-hours proliferation assay in control and in the presence of TTX 30 µM

Different ion channels are known to be transiently expressed during the cell cycle orchestrating the modulation of the resting membrane potential towards more depolarized values in G2/M and G0 phases, and more hyperpolarized RMP in G1 phase and G1/S transition [51, 52]. To assess whether Na_v_ is expressed in a cell-cycle-dependent manner, we quantified the functional Na_v_-mediated current in our GSCs lines by employing the FUCCI system, which enables cells to be differentially stained according to the cell cycle phase (see Methods). FUCCI cells turn red in the G1 phase, orange/yellow upon transitioning into S-phase, green in late S-phase, and remain green through G2-phase and mitosis. During anaphase, the green probe is degraded (Fig. 3A). Cells were visually identified based on their fluorescence positivity and Na_v_-mediated current was recorded (Fig. 3B). Intriguingly, Na_v_-mediated current was mainly detected in G2/M (green positive: 7.6 ± 2.1 pA/pF, *n* = 17/22) and G0 (Non-Labelled: 11.2 ± 1.9 pA/pF, *n* = 20/23), while it was barely detectable in G1 phase (red positive: 1.0 ± 0.3 pA/pF, *n* = 10/26) and during G1/S transition (green + red positive: 0.4 ± 0.2 pA/pF, *n* = 2/9, *p* < 0.0001, Kuskal-Wallis test, Fig. 3C).

These data suggest a cell cycle-dependent functional expression of the Na_v_ channel in GSCs. For each recorded cell, we have then correlated the Na_v_ current density with the RMP (measurements were taken at the beginning of the experiment to avoid potential run-down effects due to intracellular solution dialysis). As shown in Fig. 3D, the RMP is more depolarized for GSCs expressing higher Na_v_ current density, following a logarithmic trend (R2 = 0.79, *n* = 74). To causally link the RMP to Na_v_ current, we diluted the Na_v_ blocker QX-314 500 µM in the intracellular solution and measured Na_v_ current right after whole-cell break-in (before QX-314 dialysis could happen), then again after 5 min, allowing QX-314 action. The RMP resulted significantly hyperpolarized after QX-314 dialysis (control: -31.1 ± 4.3 mV, *n* = 7; QX-314: -35.9 ± 3.5 mV, *n* = 7; *p* < 0.015, Wilcoxon test, Fig. 3D **inset**).

Considering the correlation observed between cell cycle phases and Na_v_ functional expression, we investigated Na_v_ ability to modulate cell cycle progression in our primary cell lines. We conducted a time lapse recording of GSCs expressing the FUCCI system in control condition and in the presence of TTX 30 µM. Data were acquired at different time points over 48 h and the fluorescent labelling of the cells was monitored (Fig. 3E, F). In control conditions GSCs maintained a stable cell-cycle distribution throughout the entire recording (% of cells after 1 h: G0: 40.2 ± 2.6, G1/S: 1.2 ± 2.2, G1: 35.4 ± 3.4, G2/M: 23.2 ± 1.9, *n* = 3 replicates; % of cells after 12 h: G0: 35.5 ± 1.8, G1/S: 3.1 ± 1.2,G1: 34.4 ± 1.8, G2/M: 27.5 ± 2.1, *n* = 3 replicates; % of cells after 48 h: G0: 35.7 ± 5.4, G1/S: 7.7 ± 2.5, G1: 25.5 ± 1.4, G2/M: 25.5 ± 1.9, *n* = 3 replicates; Fig. 3F **top**). When TTX was added to the medium, the distribution of GSCs was increased in the G1 phase and G1/S transition throughout the recording, and significantly reduced in the G0 phase (% of cells after 1 h: G0: 45.8 ± 2.9, G1/S: 1.2 ± 0.2, G1: 32.7 ± 2.2, G2/M: 20.3 ± 2.7, *n* = 3 replicates; % of cells after 12 h: G0: 2.9 ± 4.1, G1/S: 14.9 ± 4.4, G1: 62.3 ± 1.3, G2/M: 19.8 ± 2.2, *n* = 3 replicates; % of cells after 48 h: G0: 15.1 ± 2.5, G1/S: 6.5 ± 0.2, G1: 37.5 ± 2.1, G2/M: 41.0 ± 2.2, *n* = 3 replicates; Fig. 3F **bottom**).

The increase in the percentage of cells in the G1 phase within the first 24 h might be due to a higher fraction of cells re-entering the cell cycle, a prolonged duration of the G1 phase, or both. To investigate this phenomenon, we analyzed the cell cycle progression exclusively for GSCs that could be continuously tracked within the same field of view (Fig. 3G). The application of TTX significantly reduced the time GSCs spent in G0 phase, compared to control condition (control: 8.7 ± 1.5 h, *n* = 12 cells; TTX: 3.6 ± 0.5 h, *n* = 14; *p* = 0.0021, Tukey’s multiple comparisons test), as well as in G2/M phase (control: 11.6 ± 1.1 h, *n* = 12 cells; TTX: 8.3 ± 2.0 h, *n* = 14; *p* = 0.0283, Tukey’s multiple comparisons test). In contrast, TTX significantly prolonged the time spent by GSCs in G1 phase (control: 7.4 ± 2.8 h, *n* = 12 cells; TTX: 17.1 ± 2.1 h, *n* = 14; *p* < 0.0001, Tukey’s multiple comparisons test). An increase in the G1 fraction of GSCs within the first 12 h and an increase in G2/M after 24 h should lead to enhanced cell proliferation. To test this hypothesis, we conducted a 96-hour proliferation assay, revealing a significant increase in cell proliferation within 48 h from TTX-induced pharmacological blockade of Na_v_ (control: 0.62 × 10^5^ number of cells at 48 h, *n* = 6; TTX: 1.98 × 10^5^ number of cells at 48 h, *n* = 6; *p* < 0.0001, Two-way ANOVA, Fig. 3H).

The data presented in this section unveil a cell cycle-dependent functional expression of the Na_v_ channel in GSCs. We observed a predominant presence of Na_v_-mediated current in G2/M and G0 phases, while it was scarcely detectable in G1 phase and G1/S transition. Pharmacological blockade of Na_v_ with TTX not only influenced the cell cycle distribution of GSCs, causing a shift towards G1 phase and G1/S transition, but also resulted in increased cell proliferation, thus emphasizing the crucial role of Na_v_ in modulating cell cycle dynamics in GSCs.

### Blockade of Na_v_ in GSCs enhances ERK1/2 and AKT signaling

It has been previously reported that in other tumors Na_v_ may influence the activity of various intracellular signaling pathways [53, 54]. To explore this possibility in GBM, we examined whether a sustained pharmacological blockade of Na_v_ and its subsequent downregulation may result in an increase of the extracellular signal-regulated kinase (ERK1/2) and PI3K/Akt/mTOR cascade, two signaling pathways notably implicated in promoting cell cycle progression and differentiation in cancer cells [55, 56].

**Fig. 4:**
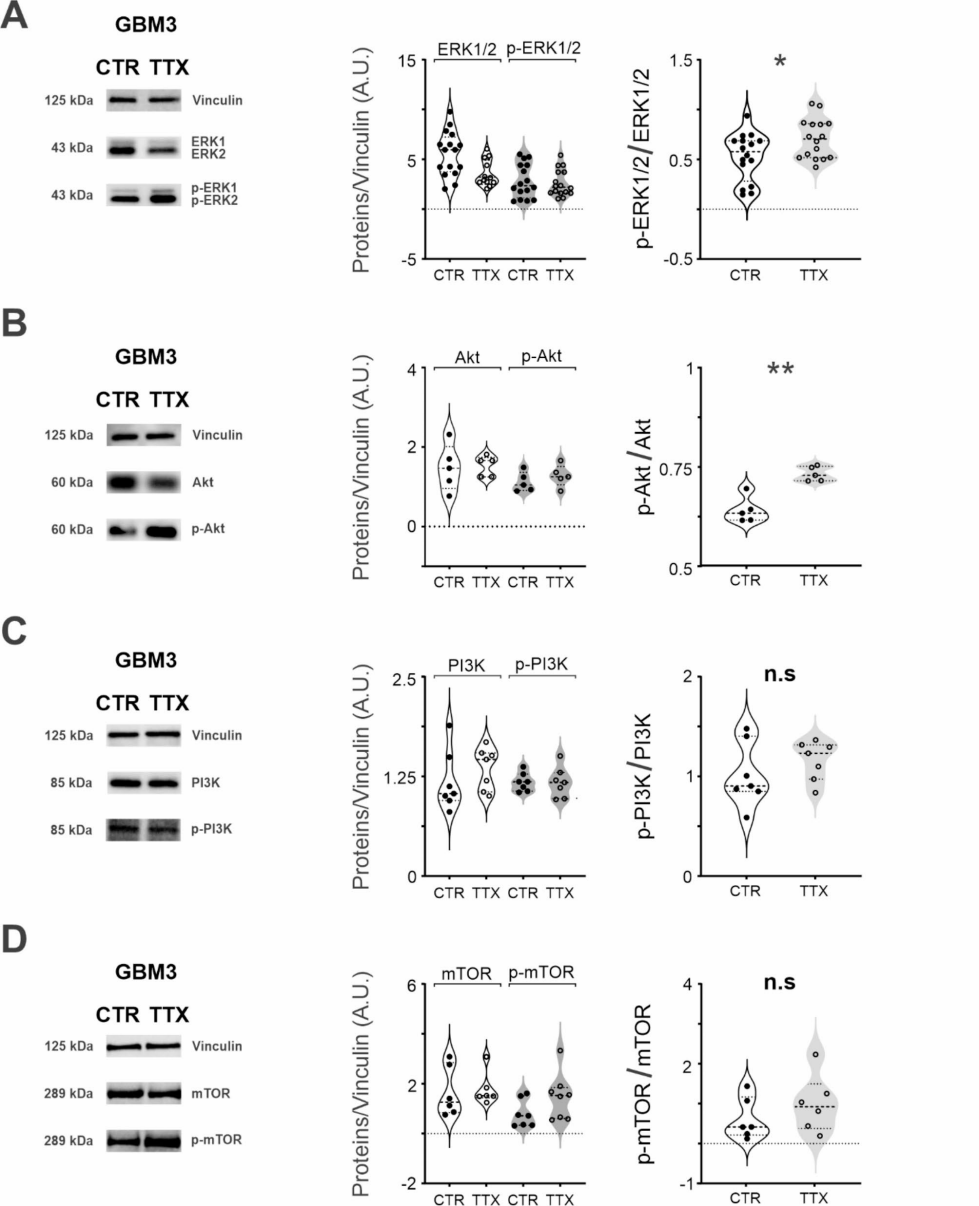
Na_v_ functional activity suppresses ERK and Akt pathway: (**A**) (left) Example of a Western Blot for total and phosphorylated p44/p42 ERK1/2 in both control and TTX condition. (center) Analysis quantification for the p44/p42 ERK1/2 and the phospho p44/p42 ERK1/2 in control and TTX condition. (right) Ratio between the p44/p42 ERK1/2 and the phospho p44/p42 ERK1/2 in control and TTX condition. (**B**)(left) Example of a Western Blot for the Akt and phospho-Akt (Ser473) in both control and TTX condition. (center) Analysis quantification for the Akt and phospho-Akt (Ser473) in control and TTX condition. (right) Ratio between the Akt and the phospho-Akt in control and TTX condition. (**C**) Example of a Western Blot for the p85 PI3K and the phospho-p85/p55 PI3K in both control and TTX conditions. (center) Analysis quantification for the p85 PI3K and the phospho-p85/p55 PI3K in control and TTX condition. (right) Ratio between the p85 PI3K and the phospho-p85/p55 PI3K in control and TTX condition. (**D**) Example of a Western Blot for the mTOR and the phospho-mTOR-Ser2448 in both control and TTX conditions. (center) Analysis quantification for the mTOR and the phospho-mTOR-Ser2448 in control and TTX condition. (right) Ratio between the mTOR and the phospho-mTOR-Ser2448in control and TTX condition

Following 72 h application of TTX 30 µM, we checked for alterations in the ERK1/2 and PI3K/Akt/mTOR cascades (Fig. 4). Our data revealed a noteworthy increase in the ratio of phosphorylated ERK1/2 (pERK1/2) on total ERK1/2 (control pERK/ERK: 0.7 ± 0.1; TTX pERK/ERK: 2.6 ± 0.3; *n* = 16; *p* = 0.0296, Mann-Whitney test; Fig. 4A, right panel) without affecting ERK1/2 and pERK1/2 total protein levels (control ERK1/2: 5.6 ± 0.6 protein/vinculin, *n* = 16; TTX ERK1/2: 3.7 ± 0.3 protein/vinculin, *n* = 16; control pERK1/2: 2.8 ± 0.4 protein/vinculin, *n* = 16; control pERK1/2: 0.52 ± 0.10, *n* = 16; Fig. 4A, central panel ), suggesting an activation of the pathway.

Similarly, we observed a significant increase in the ratio of phosphorylated Akt (p-Akt) on total Akt after 72 h treatment with TTX (control p-Akt/Akt: 0.64 ± 0.01, *n* = 5; TTX p-Akt/Akt: 0.73 ± 0.01, *n* = 5; *p* = 0.0027, Mann-Whitney test; Fig. 4B, right panel), but no difference in total protein levels (control Akt: 1.48 ± 0.26 protein/vinculin, *n* = 5; TTX Akt: 1.53 ± 0.11 protein/vinculin, *n* = 5; control p-Akt: 1.12 ± 0.11 protein/vinculin, *n* = 5; TTX p-Akt: 1.28 ± 0.12 protein/vinculin, *n* = 5; Fig. 4B, central panel). These findings suggest that Na_v_ functional expression inhibits ERK1/2 and Akt phosphorylation. Interestingly, no changes were detected either in the upstream PI3 kinase (PI3K) (control PI3K: 1.2 ± 0.1 protein/vinculin, *n* = 7; TTX PI3K: 1.4 ± 0.1 protein/vinculin, *n* = 7; control p-PI3K: 1.2 ± 0.04 protein/vinculin, *n* = 7; TTX p-PI3K: 1.2 ± 0.1 protein/vinculin, *n* = 7; control p-PI3K/PI3K 1.1 ± 0.1, *n* = 7; TTX Phospho-PI3K/PI3K: 01.2 ± 0.1, *n* = 7; *p* = 0.53, Mann-Whitney test; Fig. 4C) or in downstream mTOR kinase (control mTOR: 1.7 ± 0.4, *n* = 6; TTX mTOR: 0.8 ± 0.2, *n* = 7; control p-mTOR: 0.6 ± 0.2, *n* = 6; TTX p-mTOR: 1.5 ± 0.3, *n* = 8; control p-mTOR/mTOR: 1.0 ± 0.3, *n* = 6; *p* = 0.32 Mann-Whitney test; Fig. 4D) after 72 h TTX treatment. In conclusion, our investigation into the modulation of intracellular signaling pathways by Na_v_ channels in GBM reveals a significant impact of Na_v_ functional expression on the MAPK/ERK1/2 and PI3K/Akt/mTOR cascades. These findings shed light on the complex regulatory role of Na_v_ channels in glioblastoma, providing valuable insights for potential therapeutic intervention.

### The pharmacological blockade of Na_v_ increases GBM sensitivity to TMZ

The cumulative data presented in this study consistently substantiate our hypothesis that a temporary blockade of the Na_v_ channel contributes to diminished stemness in GSCs and increases the proportion of these cells re-entering in the cell cycle. These observations are particularly relevant, as both the extent of stemness and the duration of cells residence in the G0 phase are intricately associated with chemotherapy resistance.

**Fig. 5:**
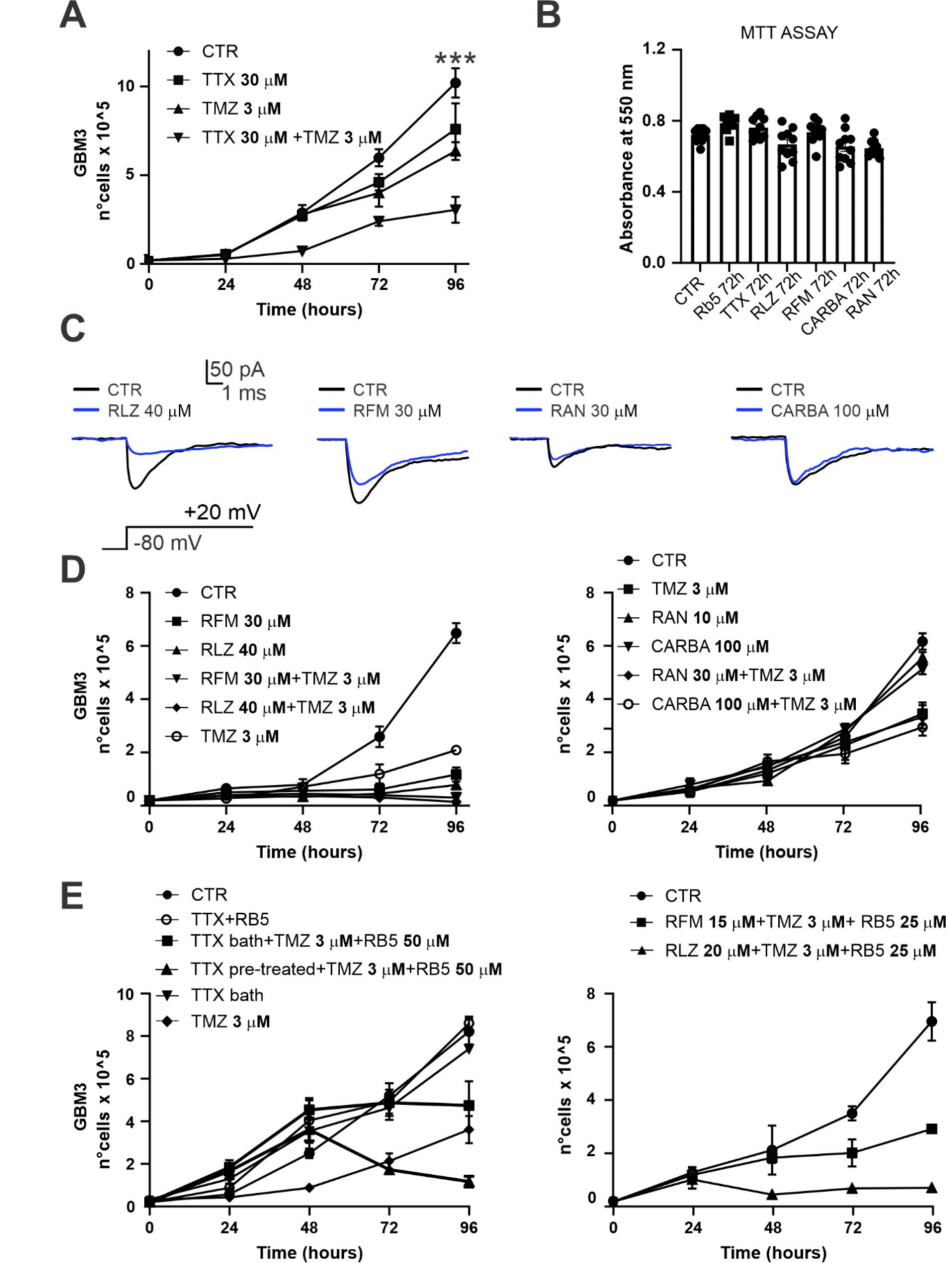
Blockade of Na_v_-mediated current increase sensibility to TMZ (**A**) GSCs proliferation assay comparing the following condition: control (control), TTX-pretreatment for 72 h (TTX), TMZ 3 µM (TMZ), and TMZ with TTX-pretreatment for 72 h (TMZ + TTX). (**B**) Effects of RB5, TTX, RLZ, RFM CARBA, RAN on cell viability/proliferation of GSCs. Data obtained with the MTT vitality assay after 72 h exposure to the displayed concentrations for each drug. (**C**) Electrophysiological example traces evoked from recorded GSCs showing the effect of RLZ, RFM, RAN, and CARBA on the Na_v_-mediated current at the indicated concentrations. The eliciting protocol is also displayed. (**D**) (left) GSCs proliferation assay comparing the effect of antiepileptic and anticonvulsant drugs that target the Na_v_ channel: control (CTR), rufinamide 30 µM (RFM), riluzole 40 µM (RLZ), and the same conditions in addition with TMZ 3 µM (RFM + TMZ; RLZ + TMZ respectively). (right) Same combinations as for (B left) but with Ranolazine (RAN 10 µM) and Carbamazepine (CARBA 100 µM). (**E**) (left) TMZ increased sensitivity mediated by Na_v_ blockade (TTX 30 µM) can be increased when combined with ERK1/2 agonist RB5 50 µM. (right) TMZ sensitivity induced by Nav blockade with RFM and RLZ can be increased when combined with ERK1/2 agonist RB5 at 25 µM

Consequently, we postulated that a pharmacological blockade of the Na_v_ channels might lead to an increased sensitivity of GBM to TMZ 3 µM treatment. To test this hypothesis, and after confirming that TTX acts exclusively on cells expressing functional Na_v_ (Supplementary Fig. 4A, B), we evaluated the growth rate of GSCs under TMZ 3 µM treatment following a 72 h pre-treatment with TTX 30 µM. After 96 h of TMZ exposure, the growth rate of GSCs was significantly reduced in cells pretreated with TTX compared to cells directly administered with TMZ alone (control: 10.2 ± 0.4 × 10^5^ cells, *n* = 4 replicates; TTX: 7.6 ± 0.7 × 10^5^ cells, *n* = 4 replicates; TMZ: 6.4 ± 0.3 × 10^5^ cells, *n* = 4 replicates; TTX + TMZ: 3.1 ± 0.3 × 10^5^ cells, *n* = 5 replicates; *p* < 0.0001, two-way ANOVA multiple comparisons, Fig. 5A). A similar effect was also observed in the U87 cell line (Supplementary Fig. 4C).

To achieve Na_v_ channel blockade using drugs more suitable than TTX for therapeutic translation, we tested clinically approved antiepileptics and anticonvulsants known to produce a negative effect on Na_v_, including riluzole (RLZ), rufinamide (RFM), carbamazepine (CARBA) and the anti-anginal ranolazine (RAN) [57]. Preliminarily, we demonstrated the lack of significant toxic effects of these drugs. In fact, RLZ (40 µM), RFM (30 µM), RAN (30 µM), and CARBA (100 µM) did not reduce cell viability more than 15% in comparison to the control condition, as assessed by MTT assay (control: 0.72 ± 0.01 a.u., *n* = 10; RB5: 0.78 ± 0.01 a.u., *n* = 10; TTX: 0.76 ± 0.02 a.u., *n* = 10; RLZ: 0.67 ± 0.03 a.u., *n* = 10; RFM: 0.74 ± 0.02 a.u., *n* = 10; CARBA: 0.66 ± 0.03 a.u., *n* = 10; RAN: 0.65 ± 0.01 a.u., *n* = 10; *p* = 0.04, Ordinary one-way ANOVA, Fig. 5B).

Electrophysiological experiments revealed a heterogeneous efficacy observed among specific antiepileptics, anticonvulsants or anti-anginals in the reduction of the Na_v_ peak current density (Fig. 5C). The different degree of reduction of Na_v_-mediated current observed among different drugs may yield distinct enhanced sensitivity to TMZ.

When applied in combination with TMZ 3 µM, both RLZ and RFM significantly reduced the growth rate of GSCs tested after 96 h, more efficiently than TMZ alone (control: 6.61 ± 0.18 × 10^5^ cells, *n* = 6 replicates; RFM: 1.15 ± 0.14 × 10^5^ cells, *n* = 6 replicates; RLZ: 0.87 ± 0.05 × 10^5^ cells, *n* = 6 replicates; TMZ: 2.16 ± 0.10 × 10^5^ cells, *n* = 6 replicates; RFM + TMZ: 0.19 ± 0.03 × 10^5^ cells, *n* = 6 replicates; RLZ + TMZ: 0.12 ± 0.05 × 10^5^ cells, *n* = 6 replicates; *p* < 0.0001, 2-way ANOVA; Fig. 5D **left**). On the contrary, neither CARBA nor RAN, alone or in combination with TMZ, exerted significantly different effects on cell growth (control: 6.45 ± 0.83 × 10^5^ cells, *n* = 3 replicates; CARBA: 6.68 ± 0.26 × 10^5^ cells, *n* = 3 replicates; RAN: 7.20 ± 0.27 × 10^5^ cells, *n* = 3 replicates; TMZ: 3.73 ± 0.17 × 10^5^ cells, *n* = 3 replicates; CARBA + TMZ: 2.83 ± 0.12 × 10^5^ cells, *n* = 3 replicates; RAN + TMZ: 3.60 ± 0.72 × 10^5^ cells, *n* = 3 replicates; *p* = 0.12, 2-way ANOVA, Fig. 5D **right**).

In Fig. 4, we showed that Na_v_ channel blockade enhances ERK1/2 activity. To further explore the molecular pathways linking Na_v_ functional activity to chemotherapy resistance, we employed a novel cell-penetrating peptide, RB5, as a functional agonist of ERK1/2 signaling [58] (Supplementary Fig. 5), that was administered alone or in addition to TMZ (3µM) following TTX pre-treatment (Fig. 5E, left). In the attempt to optimize the efficiency of TTX, we have also compared the effects of our 72 h pre-treatment to the simple addition of TTX to the medium (bath application), together with TMZ and RB5. The corresponding proliferation assay revealed increased TMZ-mediated antiproliferative efficacy when combined with TTX 72 h pre-treatment (30 µM) and RB5 (50 µM) (control: 8.21 ± 0.34 × 10^5^ cells, *n* = 3 replicates; TTX bath application: 7.52 ± 0.12 × 10^5^ cells; TTX + RB5: 8.59 ± 0.27 × 10^5^ cells, *n* = 3 replicates; TMZ: 3.84 ± 1.27 × 10^5^ cells; TTX bath + RB5 + TMZ: 4.74 ± 0.65 × 10^5^ cells, *n* = 3 replicates; TTX pre-treat + RB5 + TMZ: 1.17 ± 0.14 × 10^5^ cells, *n* = 3 replicates; *p* < 0.0001, 2-way ANOVA; Fig. 5E **left**). Intriguingly TTX pre-treatment exerts a stronger effect on GSCs proliferation in comparison to its bath application (*p* = 0.0043, Tukey’s multiple comparisons test). This combinatorial approach not only underlines the involvement of the ERK1/2 signaling cascade in the mechanism of drug resistance in GBM but also allows for a reduction in the relative drugs’ concentration to efficiently increase GSCs sensitivity to TMZ. Indeed, we were able to lower the concentration of RLZ to 20 µM and RB5 to 25 µM without diminishing their effect on GSCs growth rate when combined with TMZ 3 µM (control: 6.80 ± 0.51 × 10^5^ cells, *n* = 3; RLZ 20 µM + RB5 25 µM + TMZ: 0.55 ± 0.75 × 10^5^ cells, *n* = 3; *p* = 0.0005, Mann-Whitney test (compared to TMZ alone from Fig. 5D); Fig. 5E **right**). The effect was less pronounced when RFM concentration was decreased (RFM 15 µM + RB5 25 µM + TMZ: 2.66 ± 0.34 × 10^5^ cells, *n* = 3; *p* = 0.2, Mann-Whitney test (compared to TMZ alone from Fig. 5D); Fig. 5E **right**), with cells regaining the capability to proliferate at 96 h (Fig. 5E **right**).

Since the strongest facilitation of TMZ efficacy was observed with either TTX or RLZ, we calculated the combination index (CI) to quantitatively assess the degree of synergy between TMZ and these Nav channel modulators. Using the Chou-Talalay method, the CI values obtained were: TMZ + TTX, CI ≈ 0.64, and TMZ + RLZ, CI ≈ 0.94. These results indicate that both TTX and RLZ synergize with TMZ, enhancing its antitumor efficacy.

Taken together, these data underscore the potential therapeutic relevance of Na_v_ channel modulation, revealing a connection between Na_v_-mediated signaling pathways and increased sensitivity to chemotherapy in glioblastoma stem cells.

### The pharmacological or electrical inhibition of Na_v_ markedly impacts the proliferation of 3D GSC cultures by augmenting sensitivity to TMZ

**Fig. 6:**
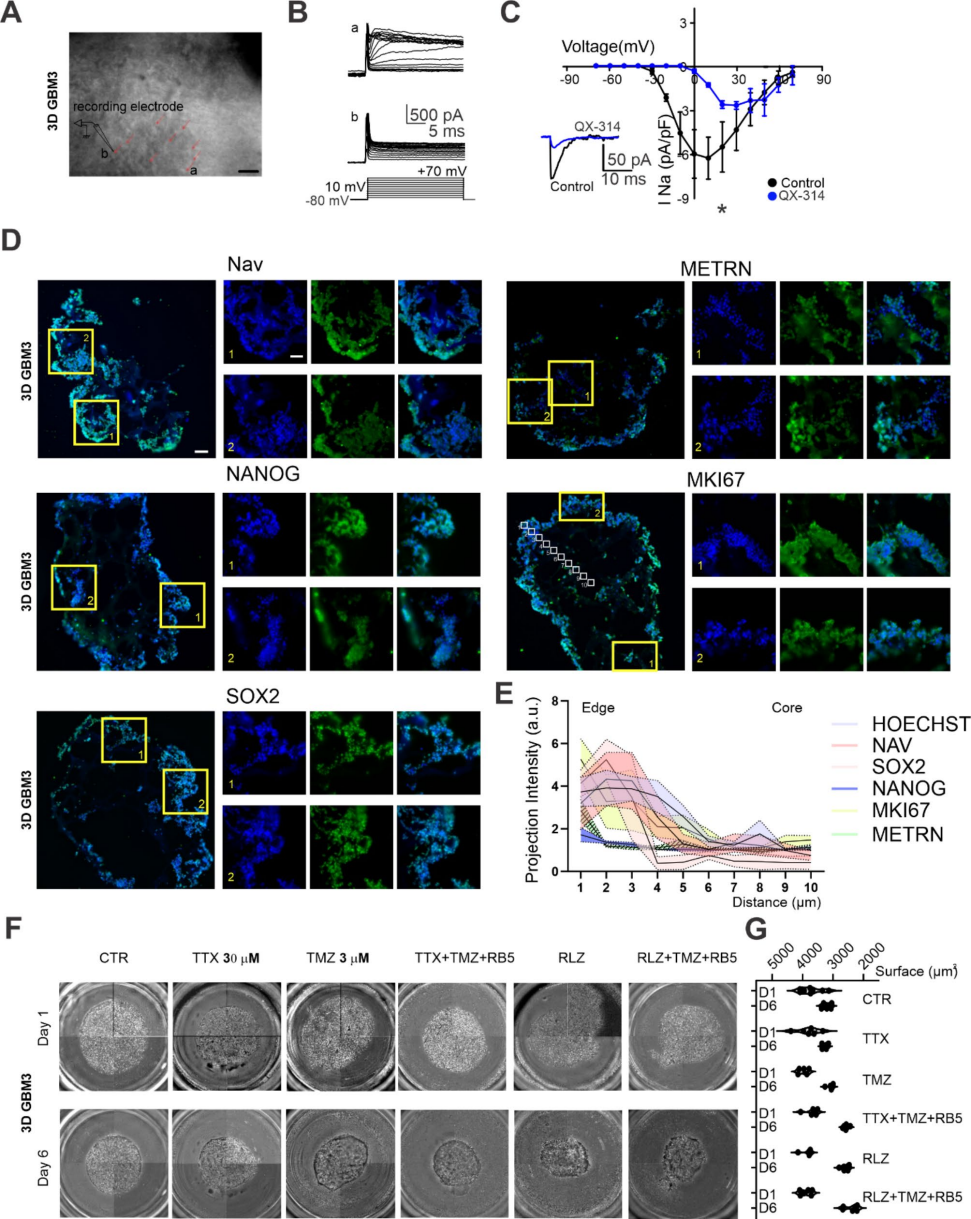
Characterization of Na_v_ expression on 3D GSC cultures and proliferation assay: (**A**) Example of the experimental approach for the electrophysiological acquisition on 3D GSCs cultures: cells somata was recognized under bright field configuration (red arrows) and approached for patch-clamp recording. a and b are two recorded cells whose traces are shown in (**B**). (**C**) Average I-V Plot of the inward current recorded in 3D GSCs cultures in control and after QX-314 intracellular dialysis. (**D**) Representative images of immunoreactivity to Pan-Na_v_ antibody, METRN, NANOG, MKI67, SOX2 (green channel) and nuclear staining (Hoechst, blue channel) for 3D GSCs cultures. Scale bars are 100 µM for low magnification pictures and 50 µM for the ROIs (**E**) Projection intensity profiles of Hoechst, Nav, SOX2, NANOG, METRN and MKI67 (each profile is an average from 3 3D GSCs cultures and 2 slices for each 3D GSCs culture).(**F**) Representative images of 3D GSC cultures at the first day of recording (Day 1),prior to treatment and after 6 days of treatment (Day 6), for the following conditions: control (CTR), TTX, TMZ, TTX + TMZ + RB5, RLZ + TMZ + RB5. Proliferation was assessed by calculating the surface of each acquired 3D GSCs cultures and averaging them for each condition. (**G**) The surface summary plot of all the conditions for Day 1 (D1) and Day 6 (D6) is displayed

In vitro 3D GBM cultures more accurately reproduce the structural characteristics of tumor tissue, including the interactions between cells and the extracellular matrix observed in vivo [28]. As a result, they offer a more precise prediction of drug efficacy compared to 2D cultures. We initially conducted electrophysiological patch clamp recordings on GSCs identified under bright field illumination (Fig. 6A), and we observed an inward current during progressively depolarizing voltage steps in 5 out of 8 recorded cells (Fig. 6B). The inward current had a reversing potential of ~ 70mV and was significantly reduced after intracellular dialysis of QX-314 through the recording electrode (control: 6.2 ± 1.4 pA/pF, *n* = 5; QX-314: 2.7 ± 0.3 pA/pF, *n* = 5; *p* = 0.03, Wilcoxon signed-rank test; Fig. 6C). The residual component of the inward current exhibited a peak at a more depolarized potential and shifted its reversing potential to the right, supporting the hypothesis that it was a voltage-dependent calcium-mediated current. To investigate the presence of Na_v_ in our 3D GSC cultures and characterize its topographical location within different compartments, we performed multiple immunoreactions for the following markers: Na_v_, SOX2, NANOG, MKI67, METRN, and the nuclear counterstaining Hoechst. After confirming the positivity of 3D GSCs cultures for all these markers (Fig. 6D, Supplementary Fig. 7), the fluorescence intensity profile was analyzed by drawing ROIs from the invading edge to the hypoxic core of the 3D GSCs cultures (as exemplified in Fig. 6D, panel MKI67). Interestingly, the localization of Na_v_ overlapped with the expression of SOX2 (Fig. 6E), confirming the co-expression previously detected in GSCs primary cell cultures (Fig. 2E).

Then, we assessed whether the block of Na_v_ would increase TMZ sensitivity in 3D GBM cultures, as we previously reported in primary GSCs (Fig. 5A). 3D GSCs culture were subjected to various treatments, including Control (CTR), TTX 30 µM, TMZ 3 µM, RLZ 20 µM, TTX 30 µM + TMZ 3 µM + RB5 25 µM, and RLZ 20 µM + TMZ 3 µM + RB5 25 µM. Daily z-stacks were acquired for a total of 36 3D cultures over 6 days and the surface areas were analyzed following a baseline acquisition on day one. Despite a gradual decrease in the surface area observed already in control condition (control day 1: 3721.0 ± 119.7 µm^2^, *n* = 8; control day 6: 3202.1 ± 47.9 µm^2^, *n* = 7; *p* = 0.006, Mann-Whitney Test; Fig. 6F, G), the most substantial reduction in comparison to the control condition at day 6 was achieved in the TTX + TMZ + RB5 (day 6: 2577.0 ± 35.2 µm^2^, *n* = 6; *p* = 0.001, Mann-Whitney Test), RLZ (day 6: 2608.7 ± 49.7 µm^2^, *n* = 6; *p* = 0.001, Mann-Whitney Test), and RLZ + TMZ + RB5 conditions (day 6: 2400.7 ± 89.0 µm^2^, *n* = 6; *p* = 0.0012, Mann-Whitney Test). However, in agreement with previous observations on primary GSCs (Fig. 2H), treatment with TTX alone resulted in a non-significant change in surface area compared to the control condition (day 6: 3269.4 ± 40.5 µm^2^, *n* = 5; *p* = 0.43, Mann-Whitney Test, Fig. 6G). It is noteworthy that the suboptimal concentration of TMZ 3 µM alone also failed to induce a significant decrease in the surface area of 3D GSC cultures compared to the control condition (day 6: 2577.0 ± 46.4 µm^2^, *n* = 5; *p* = 0.106, Mann-Whitney Test).

### Electrical activity-dependent inhibition of Nav improves sensitivity to TMZ in GSCs

In neurons, membrane conductances are often modulated by surrounding electrical activity in a frequency-dependent manner [59]. To explore whether GSCs are susceptible to activity-dependent modulation of membrane conductances, we subjected the cells to electrical stimulation at different frequencies (Supplementary Fig. 6; see Methods). After demonstrating that the density of Na_v_ can be regulated by electrical stimulation in a frequency-dependent manner (Supplementary Fig. 6B, C), we tested the sensitivity to TMZ of GSCs that were previously electrically stimulated (Supplementary Fig. 6F). According to the pharmacological experiments described above, a change in the Na_v_ current density due to a specific electrical pattern alters the TMZ sensitivity.

Taken together, these data further support our hypothesis that the negative modulation of Na_v_ functional current enhances GSCs sensitivity to TMZ, resulting in an antiproliferative effect when combined with chemotherapy.

### In vivo enhancement of Temozolomide Sensitivity in Allografted Glioblastoma Models through Sodium Channel Blockade

**Fig. 7:**
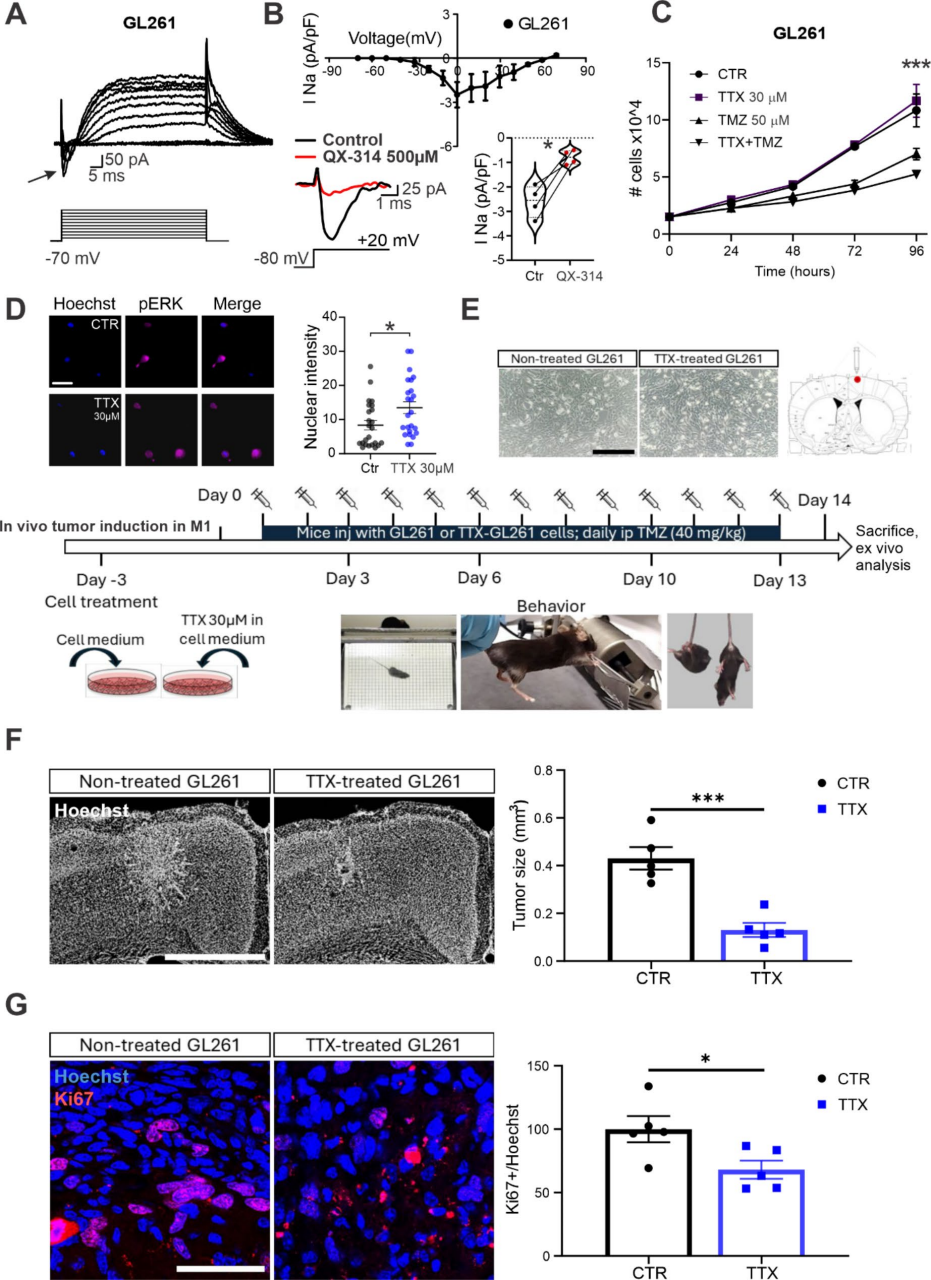
Efficacy of Temozolomide (TMZ) combine with Tetrodotoxin (TTX) in Reducing Tumor Size and Proliferation in GL261 Glioma Models: (**A**) Representative traces of inward currents recorded from GL261 cells using electrophysiological recordings. Inward currents were observed in 6 out of 11 cells tested, indicating the presence of functional Na_v_ channels. The voltage protocol used is shown below the traces. (**B**) Top: Average current density-voltage (I-V) relationship for Nav currents in GL261 cells. Representative recording on the effect of QX-314 on Na_v_ currents in GL261 cells. The inclusion of Bottom: QX-314 in the intracellular solution significantly reduced the current density of the inward current. The black trace represents control conditions, and the red trace represents the condition with QX-314. The scatter plot on the right shows pooled individual current densities in control and after QX-314 intracellular dialysis with a significant reduction observed. (**C**) Proliferation assay of GL261 cells treated with TMZ 50 µM and TTX 30 µM over 96 h. The number of cells in the control group (CTR), TTX treatment alone, TMZ treatment alone, and the combination of TTX and TMZ are displayed. A significant increase in sensitivity to TMZ was observed when combined with TTX (**D**)(left) Immunoreactivity to the phospho p44/p42 ERK1/2 antibody (magenta channel) and nuclear staining (Hoeckst, blue channel) in control condition (CTR), after 72 h TTX 30 µM treatment alone (TTX) or combined with the ERK1 blocker PD098059. Scale bar is 50 micron. (right) Phospho-ERK Optical density (OD) violin plots illustrating single-cell measurements in CTR condition and after 72 h TTX 30 µM treatment. (**E**) Schematic representation of the timeline for the in vivo study. Mice were divided into two experimental groups: one group was stereotactically injected into the primary motor cortex (M1) with untreated GL261 cells (CTR) and the other with 72 h TTX-treated GL261 cells (TTX). TMZ treatment (40 mg/kg, intraperitoneal injection) commenced the day after cell injection and was administered daily for two weeks. (**F**) Left: Representative fluorescent image of Hoechst-stained coronal brain sections from GL261 tumor-bearing mice after two weeks of treatment. Scale bar = 1 mm. Right: Pool data analysis showing that the tumor size was significantly reduced in the TTX group compared to the CTR group. (**G**) Left: Representative images of proliferating Ki67-positive cells (red) within the glioma mass (cell bodies in blue) in the motor cortex of CTR and TTX mice 14 days after glioma injection. Scale bar = 500 μm. Right: The normalized fraction of tumor area occupied by Ki67-positive cells was significantly reduced in the TTX group compared to the CTR group

To test the effect of Nav blockade in vivo, we chose to use the GL261 murine GBM cell line to inject into mice. To assess whether the GL261 cells express functional Nav-mediated currents, we conducted in vitro electrophysiological recordings. As depicted in Fig. 7A, inward currents were detected in 6 out of 11 recorded cells, indicating the presence of Nav channels in these cells. The average current density recorded in GL261 cells was 2.4 ± 0.8 pA/pF (*n* = 6, Fig. 7B). To further confirm the Nav channel-mediated nature of these currents, we dialyzed QX-314, a Nav channel blocker, into the intracellular solution of 4 of these 6 cells. The dialysis of QX-314 significantly reduced the current density of the inward current, from 2.60 ± 0.32 pA/pF under control conditions to 0.78 ± 0.14 pA/pF in the presence of QX-314 (*n* = 4, *p* = 0.0102, Wilcoxon test, Fig. 7B). Given our previous findings with the GBM3 cell line, we hypothesized that blocking Nav channels would increase the sensitivity of GL261 cells to temozolomide (TMZ). To test this, we conducted a 96-hours proliferation assay. The results revealed a significantly higher sensitivity to TMZ 50 µM when combined with TTX 30 µM, as compared to TMZ 50 µM alone (Fig. 7C). Specifically, the number of cells in the control group was 10.8 ± 0.8 × 10^4 cells (*n* = 3), while the TTX-treated group had 11.7 ± 0.8 × 10^4 cells (*n* = 3). The TMZ alone group showed a reduced cell number of 7.0 ± 0.3 × 10^4 cells (*n* = 3). Notably, the combination of TTX and TMZ further reduced cell proliferation to 5.3 ± 0.1 × 10^4 cells (*n* = 3, *p* < 0.0001, two-way ANOVA; TMZ vs. TTX + TMZ: *p* = 0.006, Multiple paired t-test). Additionally, we investigated whether TTX pre-treatment modulates the levels of phosphorylated ERK (pERK) compared to control groups. Immunostaining for pERK revealed a significant reduction in nuclear pERK intensity in the TTX pre-treated group (13.49 ± 8.4 mean nuclear fluorescent intensity per cell, *n* = 24) compared to the control group (8.347 ± 6.694 mean nuclear fluorescent intensity per cell, *n* = 24, *p* = 0.0153, Mann-Whitney test, Fig. 7D), suggesting that Nav channel blockade may reduce proliferative signaling pathways in glioma cells (Fig. 7D).

Next, we evaluated the effects of Nav blockade on tumor growth in vivo. C57BL/6J mice were divided into two groups: one group was injected with untreated GL261 cells (control group, CTR) and the other group with GL261 cells pre-treated with TTX 30 µM for 72 h. Both groups received daily TMZ treatment for 13 days following cell injection. The physiological status of the animals, including the impact of the treatment on motor function and various behavioral responses, were continuously monitored and are detailed in Supplementary Fig. 8. As shown in Fig. 7E, the experimental timeline included cell treatment, injection, and behavioral monitoring, concluding with the sacrifice and analysis of tumor size and proliferation at day 14. Tumor size was significantly reduced in the TTX pre-treated group compared to the control group, with mean tumor volumes of 0.13 ± 0.03 mm³ for the TTX group and 0.43 ± 0.05 mm³ for the control group (*n* = 5, *p* = 0.0006, unpaired t-test, Fig. 7F).

Additionally, the proliferation marker Ki67 was quantified in both groups. The normalized fraction of tumor area occupied by Ki67-positive cells was significantly lower in the TTX pre-treated group (68.02 ± 7.19) compared to the control group (100 ± 10.26), indicating reduced cell proliferation in response to TMZ treatment (*n* = 5, unpaired t-test, *p* = 0.034, Fig. 7G).

These results demonstrate that Nav channel blockade, in combination with TMZ treatment, significantly reduces glioma cell proliferation both in vitro and in vivo, enhancing the therapeutic efficacy of TMZ.

## Discussion

In the present study, GSCs along with 3D GSC cultures were employed based on their functional expression of Na_v_ channel. Major findings from the present study include: (i) the expression of Na_v_ was associated with worsened survival rate in patients with proneural subtype of GBM and a positive correlation between Na_v_ and stemness markers has been identified; (ii) pharmacological blockade of Na_v_-mediated current with TTX significantly reduced stemness markers and GSCs self-renewal capability while altering the cells cycle, promoting transition from G2/M and G0 towards G1 phase; (iii) Na_v_ pharmacological blockade causatively activated the ERK1/2 and Akt pathways and reduced expression of SOX2 and NANOG [60]; (iv) the increase in the fraction of proliferative GBM cells at the expense of the cancer stem cells population increased sensitivity to TMZ and significantly reduced the capability of GBM cells to acquire resistance to the treatment; (v) the most effective combinatorial approach for reducing GBM cell proliferation was obtained when TTX was substituted with RLZ and associated with the ERK1/2 activator RB5 in the presence of TMZ.

Na_v_ channels were originally identified in excitable cells and a total of 9 Na_v_ isoforms has been described in a variety of cell types. Interestingly, their abnormal expression has been observed in non-excitable cancer tissues like breast [18, 61], lung [62, 63], prostate [64, 65] colon [66] and cervix [67, 68], and linked to cancer cell invasion and metastasis, while these channels are absent in corresponding non-cancerous tissues.

However, the role of Na_v_ in GBM has not been fully unveiled yet. The first report available on VGSCs detected the expression of different isoforms in GBM compared to other tumors [69]. Indeed, the main isoforms involved in breast, colorectal and prostatic cancers are Na_v 1.5_, Na_v 1.7_ and Na_v 1.8_, whereas in GBM the most common isoforms expressed are Na_v 1.1_ and Na_v 1.3_ according to GlioVis data portal [44], even though other isoforms might be also significantly expressed [43]. Lately, mutations on the Na_v_ channel have been correlated with shorter survival rates in GBM patients [23].

In the present study, we have causally linked the expression of Na_v_ with the regulation of stemness in GBM via regulation of the RMP as well as the ERK1/2 signaling pathway. Shifts in the cell RMP are essential for cell cycle progression and the role of Na_v_ in this process has been only recently elucidated [17, 51]. The distinctive properties of this channel enable it to regulate the RMP in cells that are more depolarized than neurons or astrocytes, such as cancer cells. At potentials slightly more hyperpolarized than the average RMP of GBM cells (~ 45 mV), the channel is positioned at the cusp of transitioning from a closed to an open state, actively contributing to the depolarization of the cell. However, if the cell reaches excessively depolarized potentials, the Na_v_ channel undergoes inactivation, ceasing its contribution to RMP regulation. Nevertheless, when GBM cells are maintained at more hyperpolarized potentials, the density of Na_v_ is adequate to induce a rapidly inactivating depolarization resembling a ‘spikelet,’ providing direct evidence of the excitability capacity of GBM cells [70] (also refer to Supplementary Fig. 6A).

Our data show a direct effect of Na_v_ channel blockade in the transition of GSCs from depolarized phases (G2/M and G0) towards more hyperpolarized ones (G1/S transition) of the cell cycle. This evidence, together with the effects of Na_v_ current inhibition on the ERK1/2 cascade, allowed us to demonstrate that targeting this channel leads to improved sensitivity to TMZ. Based on our findings, the link between Na_v_ and the ERK1/2 and Akt pathways appears to be distinct when compared to other tumors. While our data support the hypothesis that Na_v_ activity is negatively correlated with ERK1/2 and Akt downstream signaling, previous studies have revealed an increase in MAPK signaling when Na_v_ activity is elevated [53]. This discrepancy may stem from differences in the Na_v_ subunits involved (Na_v1.1_ vs. Na_v1.5_), potential variations in mediators between the channel and the effector, or a secondary effect wherein upregulation of ERK1/2 and Akt negatively modulates Na_v1.1_ functional expression [71].

The potential regulatory roles of SCN1A/Nav1.1-interacting β subunits should be explored in depth in future studies. β subunits are crucial in modulating channel opening frequency and recruiting downstream effectors, thereby influencing signaling pathways affected by Na_v_ activity. In cancer biology, β subunits have been implicated in processes such as cell adhesion, migration, and metastasis [19, 68, 72, 73]. Understanding the interplay between SCN1A and its corresponding β subunits in GBM could provide deeper insights into the molecular mechanisms underlying GBM stemness, invasion, and resistance to TMZ, potentially unveiling novel therapeutic targets. Additionally, modulating and altering Na_v_ expression might change intracellular sodium ion dynamics, thereby affecting the function of other ion pumps such as the sodium-calcium exchanger (NCX) [30] and the sodium-hydrogen exchanger (NHE) [74, 75], which are known to play significant roles in cancer cell survival and proliferation. Ion channels regulate a variety of physiological pathways, and we cannot dismiss the potential involvement of complementary processes, such as glycolytic acidification [50], invadopodia formation and remodeling [30, 76], secretory activity [77, 78], and transcript regulation [79], as part of the antitumoral effect induced by Na_v_ blockade. The efficacy of some antiepileptics and anticonvulsants in downregulating the Na_v_-mediated current, opens the perspective for a potential therapeutic translation [80]. Considering novel groundbreaking findings regarding GBM interaction with the microenvironment, it has been recently discovered that neurons can form synaptic contacts to GBM cells [81] inducing a cascade of events with the results of a positive effect on the tumor growth [82–84]. Neuronal mechanisms can trigger GBM cell growth and invasion and sustain intratumoral cellular heterogeneity.

Given these considerations, the use of antiepileptic and anticonvulsant agents targeting the Na_v_ channel, in conjunction with chemotherapy and radiotherapy, may exert a pivotal antitumor efficacy with a potential tripartite effect: (a) mitigating seizures that afflict GBM patients, (b) inhibiting synaptic, action potential-mediated neuron-to-GBM communication, and (c) enhancing sensitivity to TMZ by reducing the GBM stemness profile through Na_v_ blockade.

While our study primarily focused on the impact and expression of Nav channels in proneural GBM, our in vivo model utilizing the GL261 cell line provides promising support for the potential therapeutic efficacy of Nav channel blockade. However, it is important to acknowledge that GL261 cells, as previously reported, more closely resemble the mesenchymal subtype of human GBM rather than the proneural subtype [85]. This raises interesting questions regarding the common mechanisms through which TTX pretreatment may exert its effects across different GBM subtypes. Mesenchymal GBM is typically characterized by a more aggressive phenotype, with enhanced invasiveness and resistance to therapy, partly driven by the activation of signalling pathways such as NF-κB, STAT3, and MAPK/ERK [86]. The modulation of the MAPK/ERK cascade, which is relevant in both mesenchymal and proneural GBM subtypes, could underlie the broad applicability of Nav channel blockade as a therapeutic strategy. In proneural GBM, Nav channels may contribute to cell proliferation and survival, while in mesenchymal GBM, they may influence invasiveness and resistance. Further research is needed to fully elucidate the specific mechanisms by which Nav channel inhibition affects these distinct GBM subtypes and to determine whether similar therapeutic benefits can be observed in other models representing the proneural and mesenchymal subtypes.

## Conclusions

In conclusion, the findings presented in this study shed a light on the pivotal role of Na_v_ channels in glioblastoma (GBM) progression and stemness regulation in vitro and in vivo. Through a comprehensive analysis of patient data and experimental models, we demonstrated a significant correlation between Na_v_ channel expression and poor prognosis in GBM patients, particularly within the proneural subtype. Importantly, the proposed therapeutic approach of combining Na_v_ channel inhibition with conventional treatments and leveraging existing antiepileptic medications presents a novel strategy with potential clinical implications. These findings not only advance our understanding of GBM pathophysiology but also offer a promising direction for the development of more effective treatment strategies against this devastating cancer.

**Abbreviations**.

## Electronic supplementary material

Below is the link to the electronic supplementary material.

Supplementary Material 1

Supplementary Material 2

## Data Availability

No datasets were generated or analysed during the current study.

## Abbreviations

GBM: Glioblastoma
**GSCs**: Patient-derived GBM Sphere-Forming Cell
**RMP**: Resting Membrane Potential
**Na**_**v**_: Voltage-Gated Sodium Channel
**VGSCs**: Voltage-Gated Sodium Channel
**ERK1/2**: Extracellular Signal-Regulated Kinases 1/2
**AKT**: Protein Kinase B
**CNS**: Central Nervous System
**TMZ**: Temozolomide
**DMEM**: Dulbecco’s Modified Eagle Medium
**RPMI-1640**: Roswell Park Memorial Institute (RPMI) Medium
**WPMY-1**: Human Prostatic Stromal Myofibroblast Cell Line
**CDT1**: Chromatin Licensing and DNA Replication Factor 1
**mKO2**: monomeric Kusabira-Orange 2
**MAG1**: monomeric Azami-Green 1
**HEK293T**: Human Embryonic Kidney 293T
**PBS**: Phosphate-Buffered Saline
**RIPA**: Radioimmunoprecipitation Assay Buffer
**CV**: Crystal Violet
**MTT**: 3-(4,5-Dimethylthiazol-2-Yl)-2,5-Diphenyl Tetrazolium Bromide
**ASIC1A**: Acid-Sensing Ion Channel 1a
**SOX2**: SRY-Box Transcription Factor 2
**SOX10**: SRY-Box Transcription Factor 10
**EPHB4**: Ephrin Type-B Receptor 4
**MKI67**: Marker Of Proliferation Ki-67
**NANOG**: Nanog Homeobox
**NMDG**: N-Methyl-D-Glucamine
**OCT4**: Octamer-Binding Transcription Factor 4
**KLF4**: Krüppel-Like Factor 4
**METRN**: Meteorin
**GFAP**: Glial Fibrillary Acidic Protein
**MTOR**: Mammalian Target of Rapamycin
**MAPK**: Mitogen-Activated Protein Kinase
**HRP**: Horseradish Peroxidase
**BSA**: Bovine Serum Albumin
**CHEK1**: Checkpoint Kinase 1
**JAG1**: Jagged Canonical Notch Ligand 1
**JAK2**: Janus Kinase 2
**MKI67**: Marker Of Proliferation Ki-67
**DAPI**: 4′,6-Diamidino-2-Phenylindole
**PI3K**: Phosphatidylinositol 3-Kinase
**BFGF**: Basic Fibroblast Growth Factor
**EGF**: Epidermal Growth Factor
**TTX**: Tetrodotoxin
**RB5**: Peptide RB5
**RLZ**: Riluzole
**RFM**: Rufinamide
**RAN**: Ranolazine
**CARBA**: Carbamazepine
**GAPDH**: Glyceraldehyde-3-Phosphate-Dehydrogenase
**SCN1A**: Sodium Channel Protein Subunit Alpha
**OS**: Overall Survival
**RA**: Retinoic Acid
**FBS**: Fetal Bovine Serum
**SDS-PAGE**: Sodium Dodecyl-Sulfate Polyacrylamide Gel Electrophoresis
**CTR**: Control
