# Supplementary material for: Modulating voltage-gated sodium channels to enhance differentiation and sensitize glioblastoma cells to chemotherapy
Source: Cell Commun Signal. 2024 Sep 9;22:434. doi: 10.1186/s12964-024-01819-z (PMC11382371; doi:10.1186/s12964-024-01819-z)
Supplement: Supplementary file 1 — Supplementary Material 1 [file 12964_2024_1819_MOESM1_ESM.pdf]

Figure panel	Test used	One or Two Sided	n	type	Descripitive data	Comments	p value	95% CI of median difference	degrees of freedom/ other	Reported in
1A	Two way ANOVA	Two sided	4 replicates	GBM3	mean ± SEM	treatment comparison	<0.0001		F (1.134, 4.536) = 1996	Results
	Tukey's multiple comparisons test					72 hours control vs. 72 hours TMZ	0.011	-0.002650 to 2.136		Results
						216 hours control vs. 72 hours TMZ	< 0.0001	-6.586 to 13.39		Results
						288 hours control vs. 72 hours TMZ	0.031	-1.291 to 4.291		Results
1B	Mann-Whitney Test	Two-tailed	10, 12	GBM3	mean ± SEM	Control 288h-TMZ 288h	0.0051			Results
1C	Wilcoxon test (signed rank test)	Two sided	8,8	GBM3	mean ± SEM	Control-TTX	0.0078			Results
1C	Wilcoxon test (signed rank test)	Two sided	8,8	GBM3	mean ± SEM	Control-QX314	0.0156			Results
1E	Mann-Whitney Test	Two-tailed	7,7	GBM3	mean ± SEM	Control 288h vs TMZ 288h Mean Fluorescence Nav	< 0.0001			Results
1E	Mann-Whitney Test	Two-tailed	90, 90	GBM3	mean ± SEM	Control 288h vs TMZ 288h Positive Cells for Nav	0.00033			Results
1E	Mann-Whitney Test	Two-tailed	90, 90	GBM3	mean ± SEM	ontrol 288h vs TMZ 288h Mean Fluorescence E-Cadher	0.08			Results
1F	Bonferroni Test		166,163,199	Human GBM sample	mean ± SD	Proneural-Mesenchimal	3.90E-15			Results
						Proneural-Classical	1.90E-03			Results
1F	Tukey's Honest Significant Difference (HSD)		166,163,199	Human GBM samples		Proneural-Classical	<0.001			Results
						Proneural-Mesenchimal	<0.001			Results
						Classical-Mesenchimal	ns			Results
1G	One-way ANOVA		7,5,8,5	Patient Derived GBN	mean ± SEM	GBM3 Suspended-GBM3Adheren-L0605-GBM19	0.002		F (2, 22) = 3.753	Results
1H	Log Rank Test		53,109	Human GBM sample	mean ± SD	SCN1A high-SCN1A low Survival Time	0.0389			Results & Graph
1I	Pearson's product-moment correlation	Two sided	163 each marker	Human GBM sample	single data	mRNA selected markers expression (log2)	<0.0001			Results in Figure
2A	Two way ANOVA	Two sided	3 replicates	GBM3	mean ± SEM	Control-TTX 5 hours	<0.0001		F (81, 245) = 14.28	Results
	Tukey's multiple comparisons test					CD34	0.015			Results
						CHEK1	0.008			Results
						JAG1	0.022			Results
						Nanog	0.004			Results
						TAZ	0.012			Results
2A	Two way ANOVA	Two sided	4 replicates	GBM3	mean ± SEM	Control-TTX 72 hours	<0.0001		F (81, 573) = 242.6	Results
	Tukey's multiple comparisons test					CD34				Results
						CHEK1				Results
						JAG1				Results
						Nanog				Results
						TAZ				Results
2B	Multiple paired t-test	Two sided	4 replicates	GBM3	mean ± SEM	Control-TTX 72 hours NANOG	0.04			Results
						Control-TTX 72 hours SOX2	0.01			Results
						Control-TTX 72 hours MKI67	0.005			Results
2C	Multiple paired t-test	Two sided	5 replicates	GBM3	mean ± SEM	Control-TTX 72 hours METRN	0.0008			Results
			6 replicates			Control-TTX 72 hours GFAP	0.0085			Results
			6 replicates			Control-TTX 72 hours β-tubulin	0.0087			Results
2D	Mann-Whitney Test	Two-tailed	100,100	GBM3	mean ± SEM	Control-TTX 72 hours OLIG4	<0.0001			Results
2F	Mann-Whitney test	Two-tailed	11,11(wells)	GBM3	mean ± SEM	Sox2 % of positive cells Control-TTX 72 hours	0.00015			Results
						NANOG % of positive cells Control-TTX 72 hours	0.029			Results
						Nav % of positive cells Control-TTX 72 hours	0.018			Results
2G	Mann-Whitney test	Two sided	18,18 (wells)	GBM3	mean ± SEM	n colonies	<0.0001			Results
	Mann-Whitney test	Two sided	11,17 (wells)	GBM3	mean ± SEM	Relative Colony Area	0.0003			Results
2H	Mann-Whitney test	Two sided	6,6 (wells)	GBM3	mean ± SEM	n neurosphere	0.0152			Results
	Mann-Whitney test	Two sided	12,6(wells)	GBM3	mean ± SEM	Relative Neurosphere Area	0.0087			Results
3C	Kruskal-Wallis test	Two sided	17,20,10,2 (rec cells)	GBM3	mean ± SEM	Nav Densitiy in FUCCI labelled GBM3	<0.0001			Results
3H	Two way ANOVA	Two sided	6	GBM3	mean ± SEM	Number of Cells Control-TTX	<0.0001		F (4, 38) = 30.05	Results
4A	Mann-Whitney test	Two-tailed	16,16	GBM3	mean ± SEM	pERK/ERK Control-TTX	0.0296			Results
4B	Mann-Whitney test	Two-tailed	5,5	GBM3	mean ± SEM	pAkt/Akt Control-TTX	0.0027			Results
4C	Mann-Whitney test	Two-tailed	7,7	GBM3	mean ± SEM	pPI3K/PI3K Control-TTX	0.53			Results
4D	Mann-Whitney test	Two-tailed	7,7	GBM3	mean ± SEM	p-mTOR/mTORControl-TTX	0.32			Results
5A	Two way ANOVA	Two sided	4 replicates	GBM3	mean ± SEM	Proliferation assay Control-TTX-TMZ-TTX+TMZ	<0.0001		F (4, 67) = 547.1	Results
	Tukey's multiple comparisons test					TMZ-TTX+TMZ	<0.0001	24.41 to 41.79		Results
5B	Two way ANOVA	Two sided	10 replicates	GBM3	mean ± SEM	MTT Control-Rb5-TTX-RLZ-RFM-CARBA-RAN	0.04		F (6, 63) = 1.463	Results
5D	Two way ANOVA	Two sided	6 replicates	GBM3	mean ± SEM	feration assay Control-RFM-RLZ-TMZ+RFM+TMZ-RLZ+	<0.0001		F (5, 164) = 19.63	Results
5E	Two way ANOVA	Two sided	3 replicates	GBM3		Proliferation assay Control-TTX+RB5-TTX bath-TTX pretreated +TMZ	<0.0001		F (5, 48) = 55.43	Results
5E	Mann-Whitney test	Two sided	3 replicates	GBM3	mean ± SEM	RLZ+RB5+TMZ-RLZ+TMZ	0.0005			Results
6C	Wilcoxon test (signed rank test)	Two sided	5,5	Organoids	mean ± SEM	Control-Qx314 GBM organoids	0.03			Results

**6G**	Mann-Whitney test	Two sided	8,7		Organoids	mean ± SEM	Control day1-day6	0.006			Results
			8,6				TTX + TMZ + RB5 day1 -day6	0.001			Results
			8,6				RLZ + TMZ + RB5 day1-day6	0.0012			Results
**7B**	Wilcoxon test (signed rank test)	Two sided	4,4		GL261	mean ± SEM	Control-TTX Nav densitiy acute perfusion GL261	0.0102			Results
**7C**	Two way ANOVA	Two sided	3 replicates		GL261	mean ± SEM	Control-TTX-TMZ-TTX+TMZ	<0.0001		F (1.383, 11.06) = 371.2	Results
	Tukey's multiple comparisons test						TMZ-TTX+TMZ	0.006	0.1707 to 3.329		Results
**7D**	Mann-Whitney test	Two sided	24,24		GL261	mean ± SEM	Control-TTX	0.0153			Results
**7F**	unpaired t-test	Two sided	5,5	mice		mean ± SEM	Tumor Size Control-TTX-pretreated	0.0006			Results
**7G**	unpaired t-test	Two sided	5,5	mice		mean ± SEM	Ki-67 expression Control-TTX-pretreated	0.034			Results
