# Supplementary material for: Modulating voltage-gated sodium channels to enhance differentiation and sensitize glioblastoma cells to chemotherapy
Source: Cell Commun Signal. 2024 Sep 9;22:434. doi: 10.1186/s12964-024-01819-z (PMC11382371; doi:10.1186/s12964-024-01819-z)
Supplement: Supplementary file 2 — Supplementary Material 2 [file 12964_2024_1819_MOESM2_ESM.docx]

1. **Additional files:**


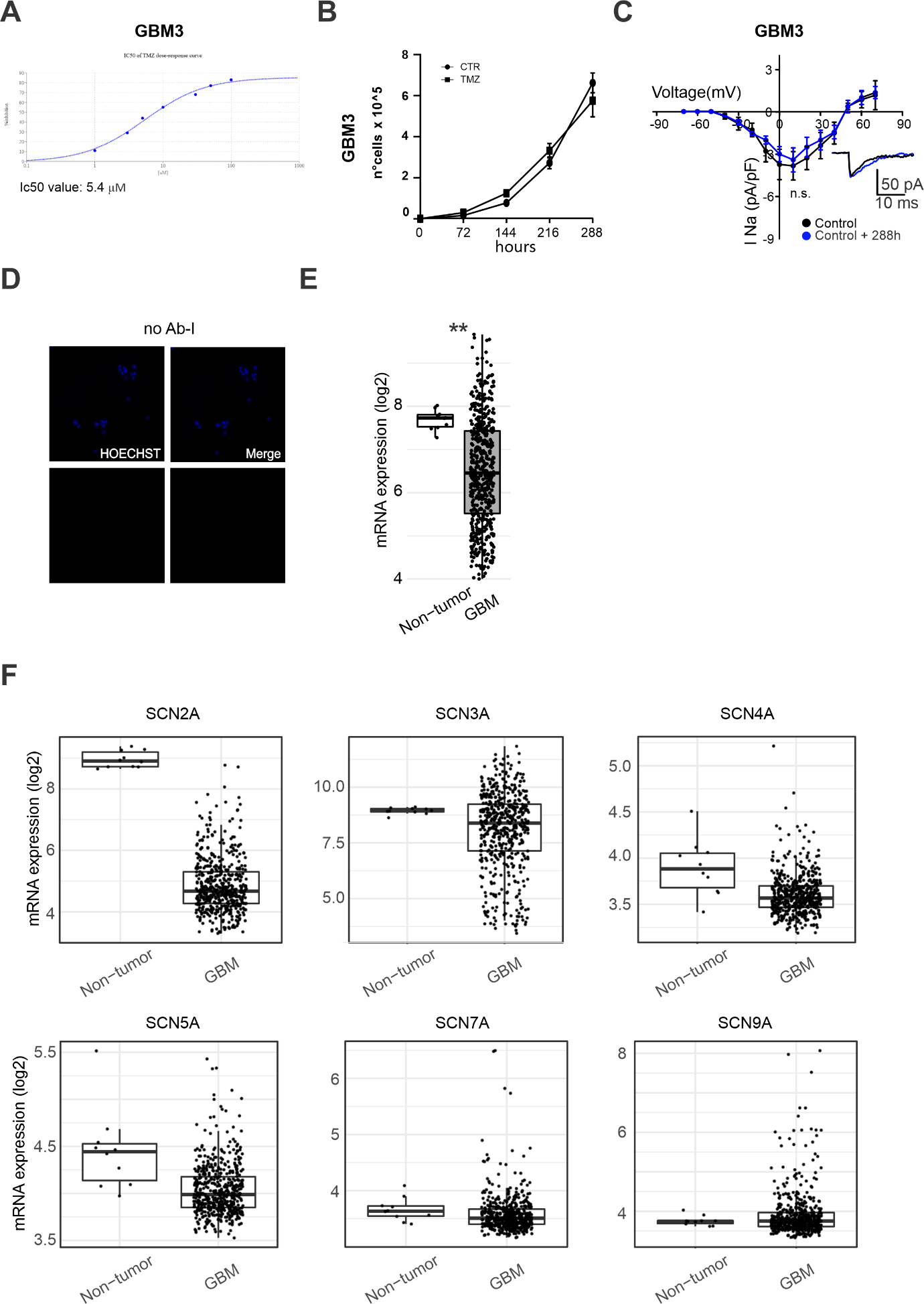


**Supplementary Figure 1 (referring to Fig.1): Na_v_ upregulation is caused by TMZ application, and it correlates with resistance induction.** (A) TMZ IC_50_ on GBM3 was calculated and plotted. The IC50 value corresponded to 5.4 μM. (B) Pool data for the 288 hours proliferation assays of proneural GSCs in control condition (circles) and in the presence of TMZ 3μM (squares) for the death number of cells. The death number of cells is significantly higher in the TMZ condition from 72 to 216 hours (72 hours: control: 0.16 ± 0.02 x 105 number of death cells; TMZ: 0.30 ± 0.08 x 105 number of death cells; n = 4; p = 0.008, Two-Way Anova Multiple Comparisons; 216 hours: control: 2.71 ± 0.17 x 105 number of death cells; TMZ: 3.3 ± 0.20 x 105 number of death cells; n = 4; p = 0.013, Two-Way Anova Multiple Comparisons; Fig. 1A, inset). At 288 hours the situation was reversed. (C) Na_v_-mediated current density is compared from recorded GSCs at time 0 (control) and after 288 hours. No significant difference in the current density was detected (control: 3.8 ± 1.0 pA/pF, n = 6; 288h: 3.4 ± 0.8 pA/pF, n = 5; p = 0.92, unpaired t-test). (D) To assess potential background, a negative control involves incubating the cells with the secondary antibody alone, excluding the primary antibody, to eliminate non-specific staining.

(E) SCN1A mRNA expression levels in brain tissues of non-tumor and GBM patients were compared (data taken from GlioVis TCGA_GBM repository).

(F) mRNA expression (log2) for the following SNC isoforms: SCN2A (GBM: 4.9 ± 0.88 log2, n= 528); SCN3A: (GBM: 8.1 ± 1.8 log2, n= 528); SCN4A: (GBM: 3.6 ± 0.2 log2, n= 528); SCN5A: (GBM: 4.0 ± 0.3 log2, n= 528); SCN7A: (GBM: 3.6 ± 0.3 log2, n= 528); SCN8A: (GBM: 3.9 ± 0.2 log2, n= 528); SCN9A: (GBM: 3.9 ± 0.6 log2, n= 528).


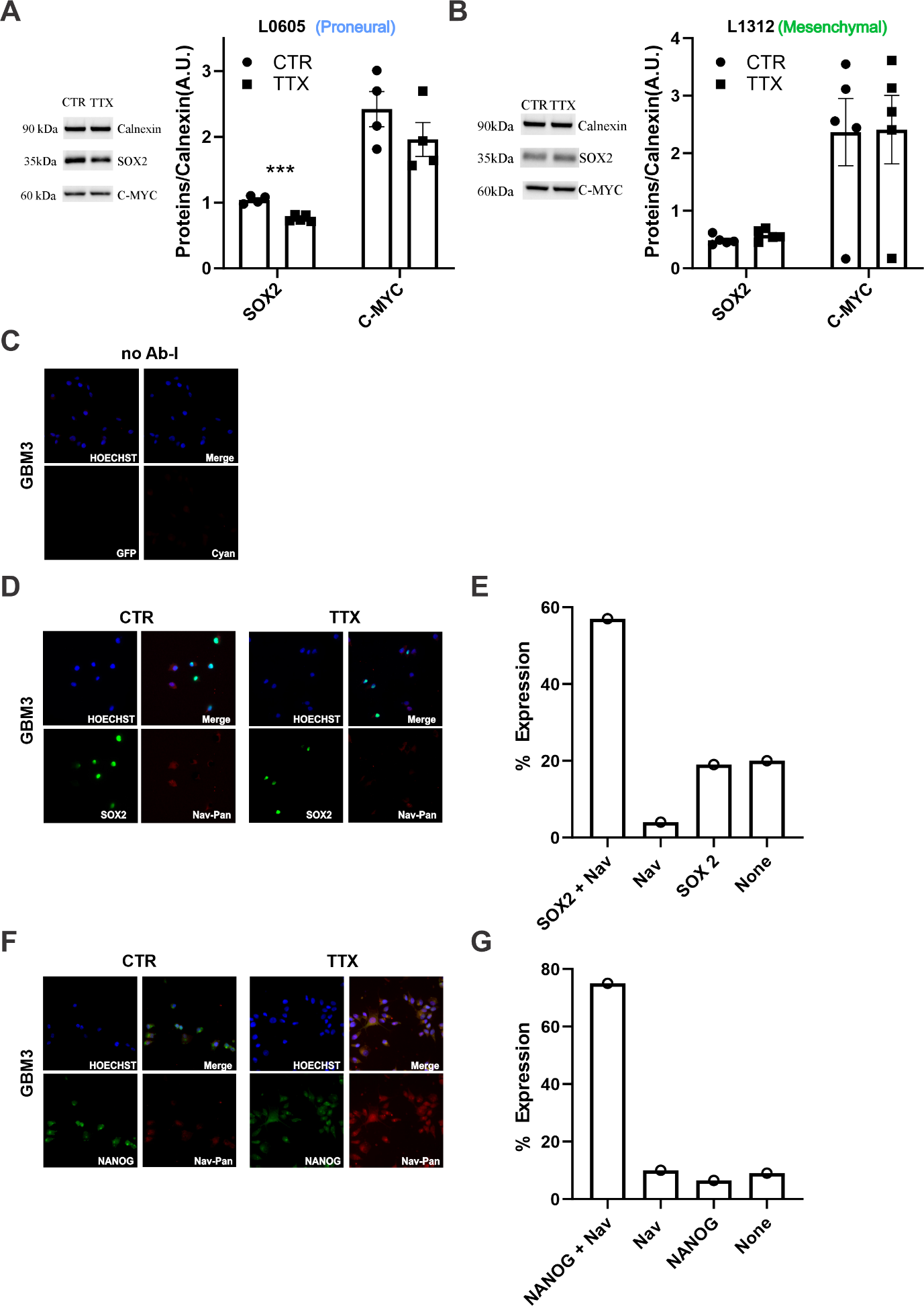


**Supplementary Figure 2 (referring to Fig.2): SOX2 is significantly downregulated in Proneural GSCs but not in mesenchymal GSCs and a high rate of co-expression between Na_v_ and SOX2 or NANOG is detected.**

(A) Western blot quantification for stemness markers reveals a significant reduction in the protein content for SOX2 but not C-MYC in a different Proneural GSCs line (L0605) when Na_v_ is blocked for 72 hours. (SOX2 control: 1.04 ± 0.03 a.u., n= 4; SOX2 TTX: 0.76 ± 0.02 a.u., n= 4; p= 0.000093 Multiple paired t-test; C-MYC control: 2.42 ± 0.27 a.u., n= 4; C-MYC TTX: 1.96 ± 0.26 a.u., n= 4; p= 0.261 Multiple paired t-test); (B) The same protein content quantification reveals no significant reduction after 72 hours of TTX treatment in a mesenchymal GSCs line (L1312). (SOX2 control: 0.49 ± 0.06 a.u., n= 5; SOX2 TTX: 0.58 ± 0.04 a.u., n= 5; p= 0.14 Multiple paired t-test; C-MYC control: 2.37 ± 0.59 a.u., n= 5; C-MYC TTX: 2.41 ± 0.60 a.u., n= 5; p= 0.261 Multiple paired t-test).

(C) To assess potential background, a negative control consists in incubating the cells with the secondary antibody alone, excluding the primary antibody, to eliminate non-specific staining.

(D) Another example of Immunoreactivity to Pan-Na_v_ antibodies (red channel), SOX2 (green channel) and nuclear staining (Hoechst, blue channel). (E) Total percentage of cells positive to SOX2 and Na_v_ (n = 54/95; 57%), only Na_v_ (n = 4/95; 4%), only SOX2 (n = 18/95; 19%) or neither of these (n = 19/95; 20%). (F) An additional instance of immunoreactivity featuring Pan-Na_v_ antibodies (red channel), NANOG (green channel), and nuclear staining (Hoechst, blue channel) (G) Total percentage of cells positive to NANOG and Nav (n = 69/92; 75%), only Nav (n = 9/92; 10%), only NANOG (n = 6/92; 6.5%) or neither of these (n = 8/92; 9%).


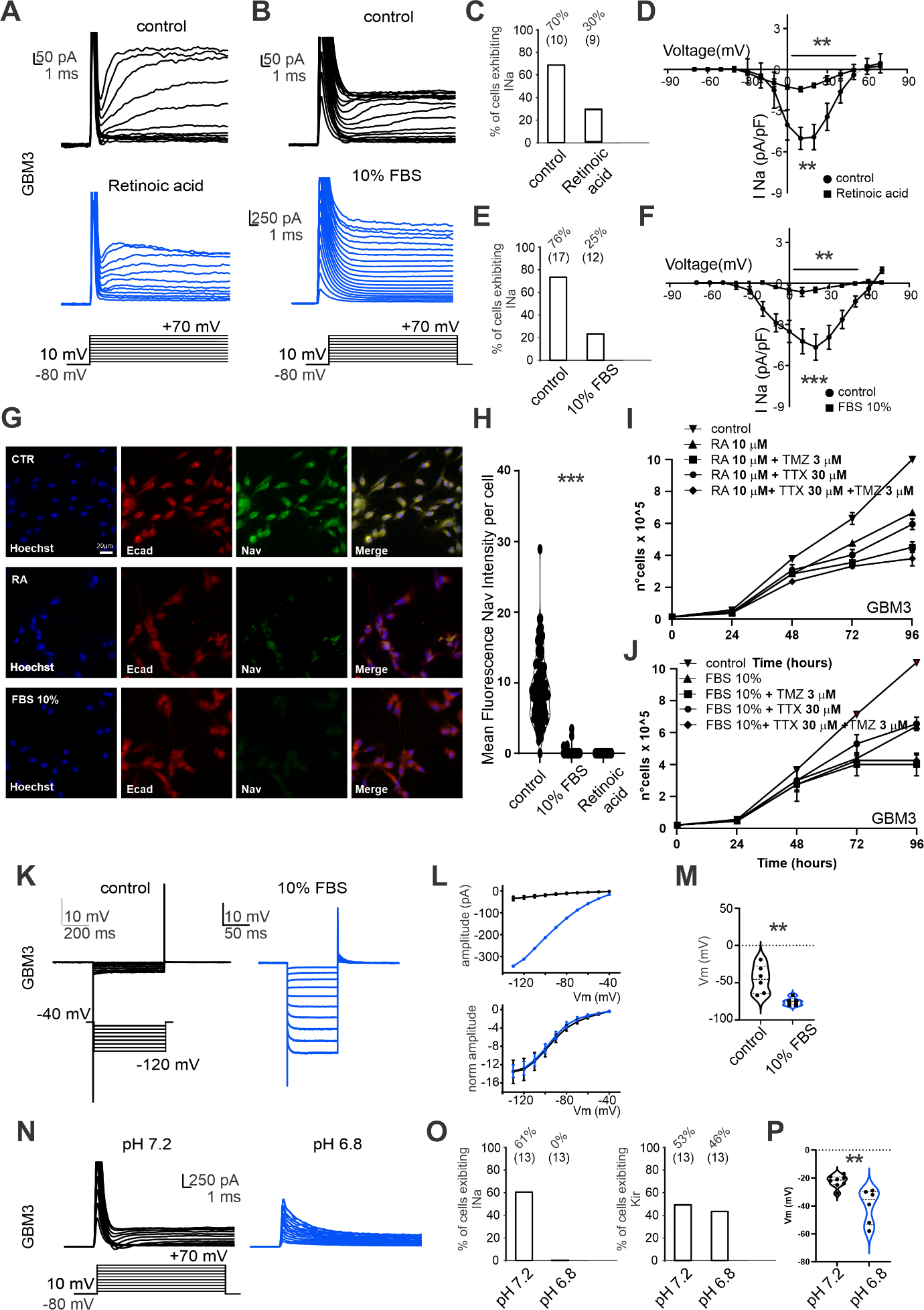


**Supplementary Figure 3:**

**The functional expression of Nav channels is markedly diminished in differentiated cells or under conditions of extracellular acidification, impacting the response to TMZ:** (A) Representative depolarizing voltage clamp traces from GSCs in control (black) and with retinoic acid (RA; 10 μM) in the medium for 5 days (blue). (B) Representative depolarizing voltage clamp traces from GSCs in control (black) and with 10% FBS in the medium for three days (blue). (C) Percentage of the cells expressing functional Nav-mediated current in control compared to RA condition (control: 7/10 cells; RA: 3/9 cells). (D) I-V plot of the Na_v_-mediated current density in control and after 48 hours RA showing a significant decrease in the density of the current (control: 5.0 ± 0.8 pA/pF, n = 10; RA: 1.4 ± 0.2 pA/pF, n = 9; p = 0.002, unpaired t-test). (E) Percentage of the cells expressing functional Na_v_-mediated current in control and with 10% FBS in the medium for 6 days revealed a reduced fraction of cells with Na_v_-mediated current (control: 7/10 cells; 10% FBS: 3/9 cells). (F) I-V plot of the Na_v_-mediated current in control and in 10% FBS condition with a decrease in current density (control: 4.7 ± 0.9 pA/pF, n = 17; FBS 10%: 0.7 ± 0.3 pA/pF, n = 12; p = 0.002, unpaired t-test). (G) Immunoreactivity to the Pan-Nav antibody (green channel), E-Cadherin antibody (red channel), and nuclear staining (Hoechst, blue channel) in control condition (CTR), after 5 days of RA (10 μM) treatment, and after 6 days of 10% FBS treatment. Scale bar is 50 μm. (H) Optical density (OD) violin plots illustrating single-cell measurements in CTR condition (OD CTR: 9.178 ± 0.4824), after 5 days of RA (10 μM) treatment (OD RA:0.3 ± 0.1), and 6 days of 10% FBS treatment (OD FBS = 0.09724 ± 0.05378) (p < 0.0001; Ordinary One-Way Anova). (I) GSCs proliferation assay comparing the following conditions: control, RA 10 μM, RA 10 μM with TTX 30 μM (RA 10 μM + TTX 30 μM), RA 10 μM with TMZ 3 μM (RA 10 μM + TMZ 3 μM), and RA 10 μM with TTX 30 μM and TMZ 3 μM (RA 10 μM + TTX 30 μM + TMZ 3 μM). RA was pretreated for 5 days in all conditions. TTX was pretreated for 72 hours in all conditions. After 96 hours of TMZ exposure, the growth rate of GSCs conditioned with RA (10 μM) was not significantly different in cells pretreated with TTX compared to cells administered with TMZ alone (control: 10.5 ± 0.1 x 10^5 cells, n= 3 replicates; RA 10 μM: 7.0 ± 0.1 x 10^5 cells, n= 3 replicates; RA 10 μM + TMZ 3 μM: 4.7 ± 0.3 x 10^5 cells, n= 3 replicates; RA 10 μM + TTX 30 μM: 6.3 ± 0.3 x 10^5 cells, n= 3 replicates; RA 10 μM + TTX 30 μM + TMZ 3 μM: 4.0 ± 0.3 x 10^5 cells, n= 3 replicates; p< 0.0001, two-way ANOVA multiple comparison; p= 0.72, Tukey's multiple comparisons test between RA 10 μM + TMZ 3 μM and RA 10 μM + TTX 30 μM + TMZ 3 μM). (J) GSCs proliferation assay comparing the following conditions: control, 10% FBS, 10% FBS with TTX 30 μM (10% FBS + TTX 30 μM), 10% FBS with TMZ 3 μM (10% FBS + TMZ 3 μM), and 10% FBS with TTX 30 μM and TMZ 3 μM (10% FBS + TTX 30 μM + TMZ 3 μM). In all of the conditions, 10% FBS was pretreated for 6 days. TTX was pretreated for 72 hours in all conditions. After 96 hours of TMZ exposure, the growth rate of GSCs conditioned with 10% FBS was not significantly different in cells pretreated with TTX compared to cells administered with TMZ alone (control: 10.4 ± 0.1 x 10^5 cells, n= 3 replicates; 10% FBS: 6.4 ± 0.1 x 10^5 cells, n= 3 replicates; 10% FBS + TMZ 3 μM: 4.0 ± 0.5 x 10^5 cells, n= 3 replicates; 10% FBS + TTX 30 μM: 6.6 ± 0.3 x 10^5 cells, n= 3 replicates; 10% FBS + TTX 30 μM + TMZ 3 μM: 4.3 ± 0.3 x 10^5 cells, n= 3 replicates; p< 0.0001, two-way ANOVA multiple comparison; p= 0.83, Tukey's multiple comparisons test between 10% FBS + TMZ 3 μM and 10% FBS + TTX 30 μM + TMZ 3 μM). (K) Representative traces recorded in voltage-clamp mode with progressive hyperpolarizing steps in control (black) and after 72 hours of 10% FBS treatment (blue). (L) (top) Plot for the average value of the steady current recorded at each hyperpolarizing step for both the control and FBS condition. (bottom) The amplitude of the steady current has been normalized to compare the activation curve between the two conditions. (M) Resting membrane potential for all the recorded cells in control and with FBS treatment. (N) Representative depolarizing voltage clamp traces from GSCs in control (black) and after bath perfusion of medium with modified pH to 6.8 (blue). (O) (Left) Percentage of the cells expressing functional Na_v_ -mediated current in control and after bath perfusion (5 mins) of medium with modified pH to 6.8 (pH 7.2: 8/13 cells; pH 6.8: 0/13 cells). (Right) Percentage of cells expressing inward rectifier current in control and after bath perfusion of medium with modified pH to 6.8. (P) RMP measurements in control and after bath perfusion of medium with modified pH to 6.8 (pH 7.2: -22.4 ± 1.5 mV, n = 6; pH 6.8: -40.0 ± 5.0 pA/pF, n = 8; p = 0.0027, unpaired t-test).


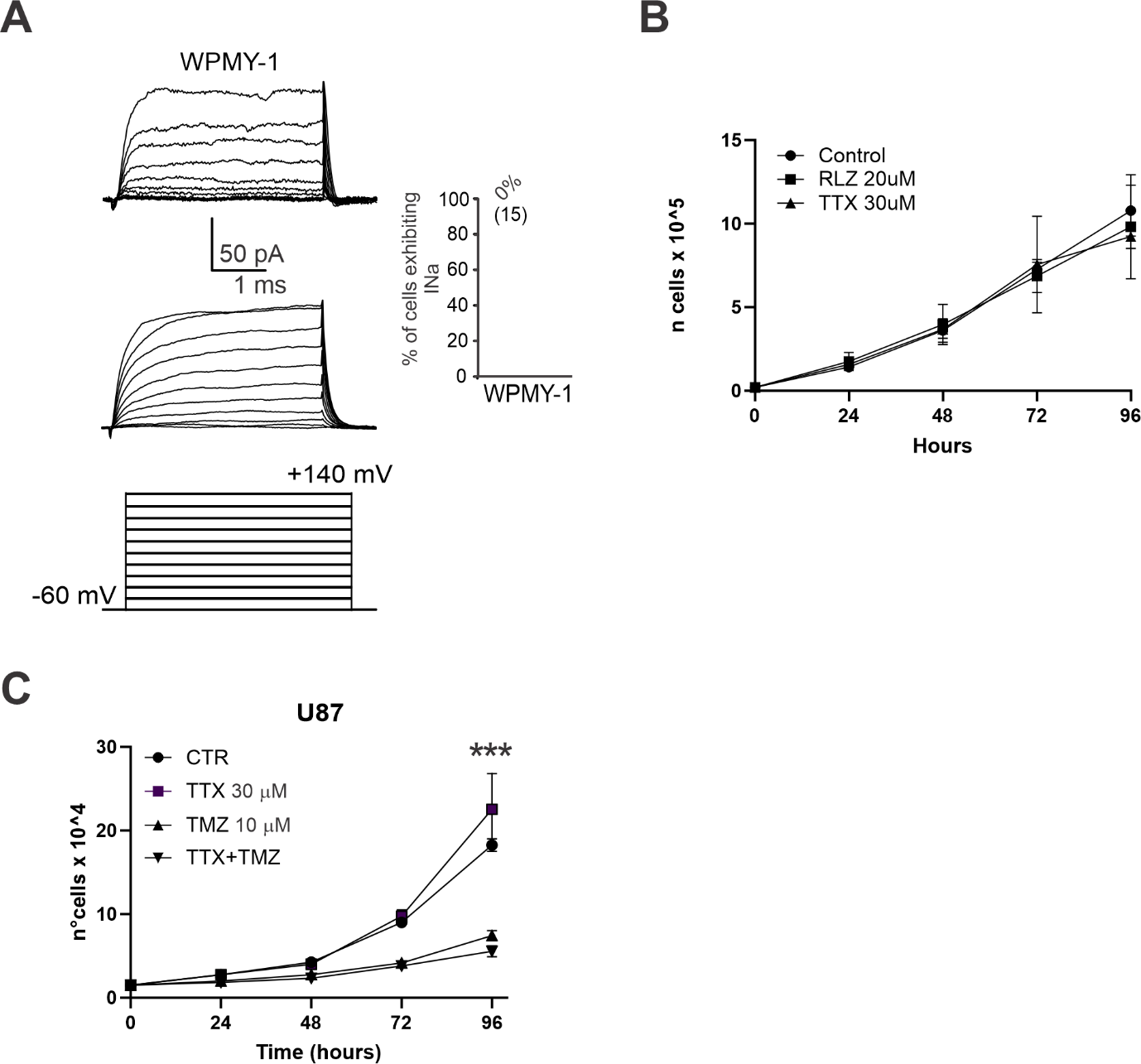


**Supplementary Figure 4 (referring to Fig. 5): TTX has no effect on cell proliferation or TMZ sensitivity when Nav is not functionally expressed, as in prostatic fibroblasts, but increases TMZ sensitivity in the U87 GBM cell line.** (A) The absence of functional Na_v_ expression was tested in a prostatic fibroblasts cell line (WPMY-1) with the whole-cell patch clamp approach (the protocol is displayed at the bottom of the panel) confirming the previous literature [69] (cell expressing functional inward current n = 0 out of 15). Two recorded WPMY-1 cells are provided (B) Proliferation assay (96 hours) of the WPMY-1 line in control (96 hours: control: 10.7 ± 0.76 x 10^5^ cells, n = 4; RLZ 20 μM: 9.82 ± 1.56 x 10^5^ number cells; n = 4; RLZ 30 μM: 9.24 ± 0.41 x 10^5^ cells, n = 3; p = 0.9967, Two-Way Anova). (C) We wanted to test whether blocking Nav channels could also increase sensitivity to TMZ in an immortalized GBM cell line, such as U87 [43]. To assess this, we conducted a 96-hour proliferation assay. The results revealed a significantly higher sensitivity to TMZ 10 μM when combined with TTX 30 μM, as compared to TMZ 10 μM alone. Specifically, the number of cells in the control group was 18.25 ± 0.43 x 10^4 cells (n=3), while the TTX-treated group had 22.53 ± 2.51 x 10^4 cells (n=3). The TMZ alone group showed a reduced cell number of 7.43 ± 0.35 x 10^4 cells (n=3). Notably, the combination of TTX and TMZ further reduced cell proliferation to 5.55 ± 0.375 x 10^4 cells (n=3, p<0.0001, two-way ANOVA; TMZ vs. TTX + TMZ: p=0.0039, Multiple paired t-test).


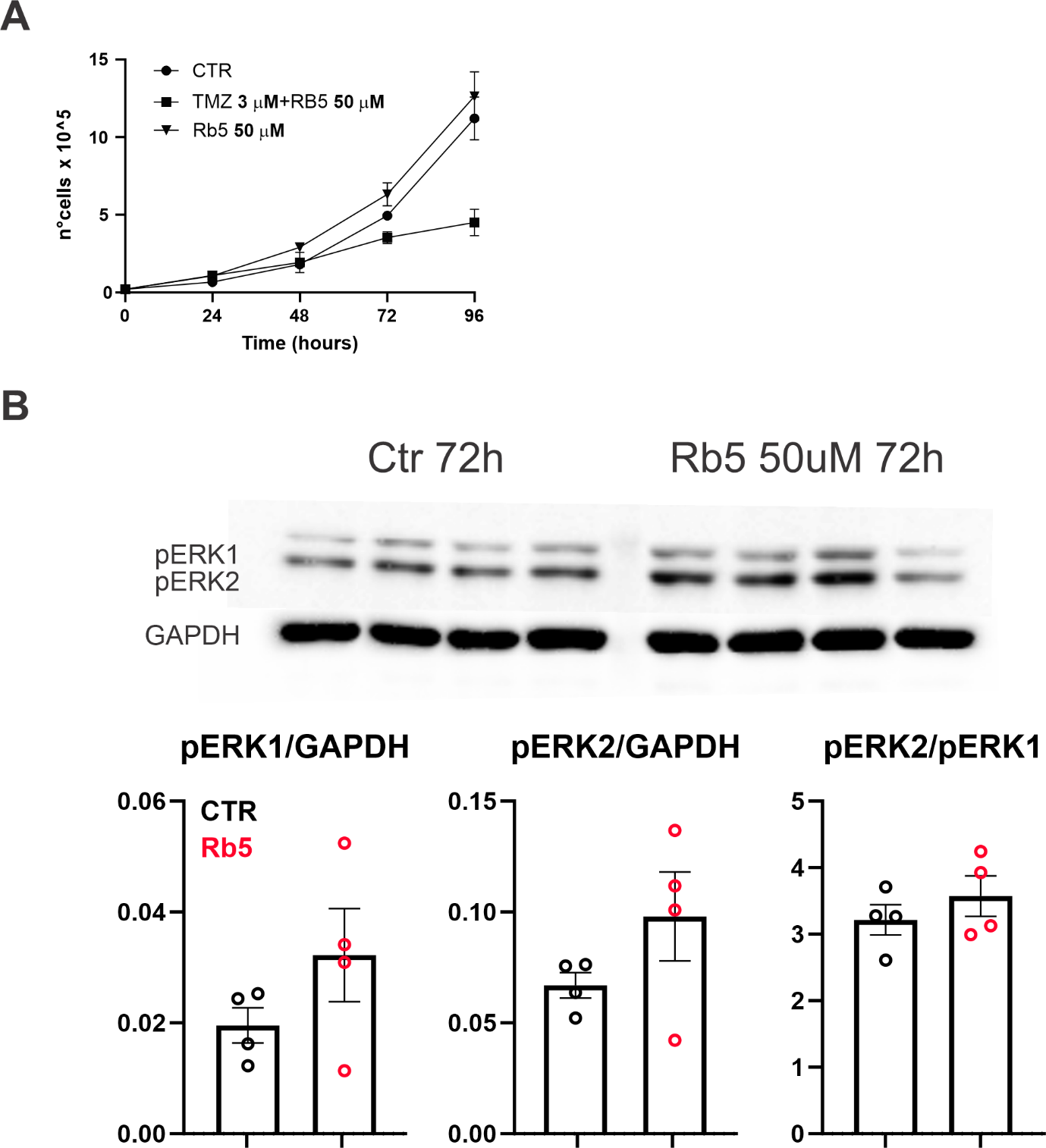


**Supplementary Figure 5 (referring to Fig.5): Effect of RB5 on GSCs**

(A) Proliferation assay (96 hours) of GSCs in control (96 hours: control: 11.2 ± 0.78 x 10^5^ cells, n = 3; TMZ: 4.51 ± 0.49 x 10^5^ number cells; n = 3; RB5: 11.7 ± 0.33 x 10^5^ cells, n = 3; p < 0.0001, Two-Way Anova). (B) Western blot analysis reveals an activation of both phosphorylated ERK1 (control: 0.019 ± 0.003 pERK1/GAPDH, n = 4; RB5: 0.032 ± 0.008 pERK1/GAPDH, n = 4) and phosphorylated ERK2 (control: 0.067 ± 0.006 pERK2/GAPDH, n = 4; RB5: 0.098 ± 0.02 pERK2/GAPDH, n = 4) when RB5 (50 μM) is present in the medium for 72 hours, compared to control condition (CTR).


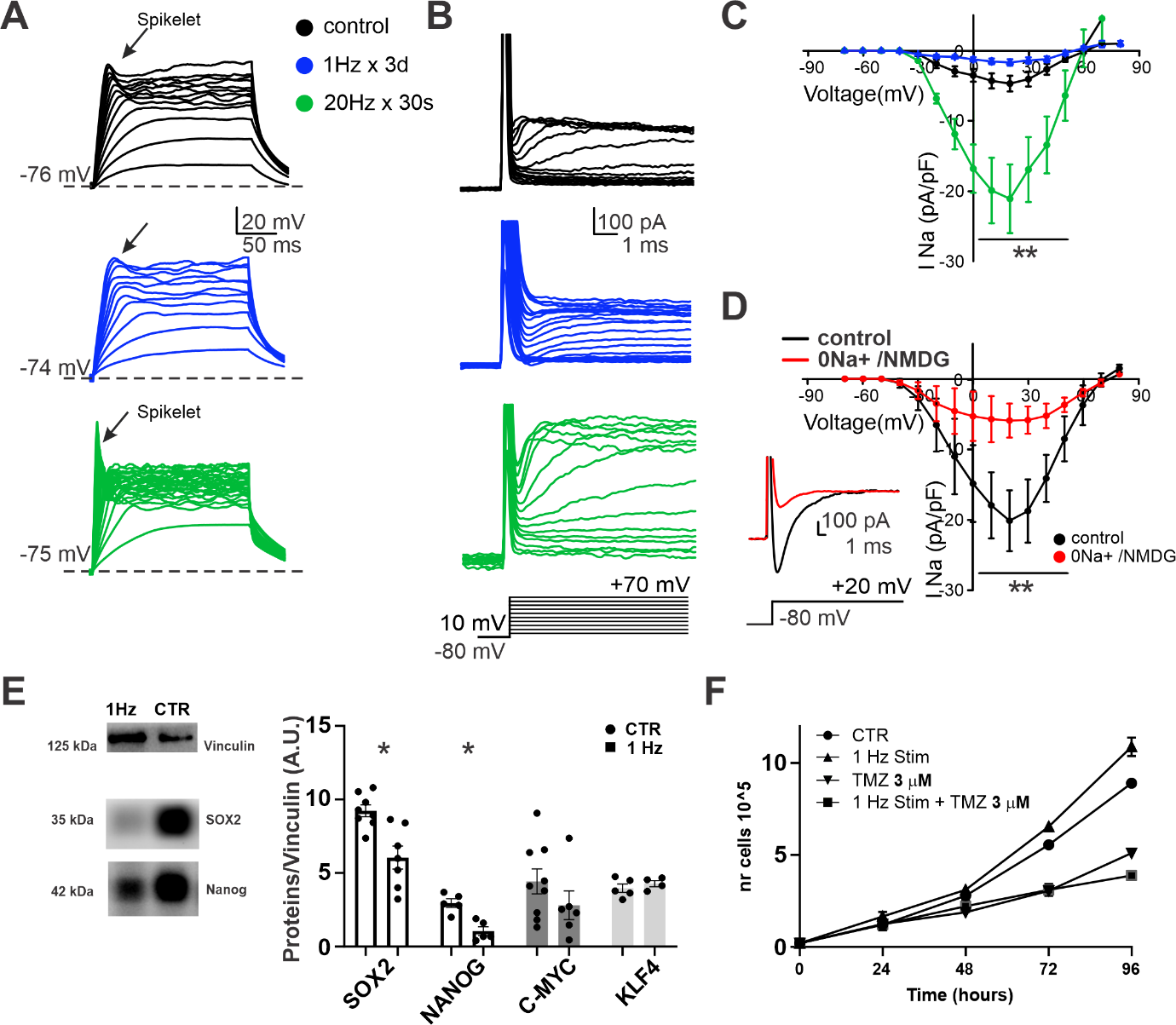


**Supplementary Figure 6: Activity-dependent regulation of Na_v_ functional density:** (A) Representative current-clamp (left) and voltage-clamp (right) recordings from GSCs recorded in control condition (black), electromagnetically stimulated with a frequency of 1 Hz (blue) and 20Hz (green). In current clamp, GBM RMP was held at ~-75 mV. (C) Pool data of the I-V curve obtained from voltage-clamp recordings for all stimulation frequencies shown in A. Stimulation at 20 Hz resulted in a significant upregulation of functional Na_v_-mediated current density. Conversely, prolonged 1 Hz stimulation led to a pronounced downregulation of Na_v_ functional current density (control: 4.0 ± 0.8 pA/pF, n = 6/17; 20 Hz: 19.8 ± 3.3 pA/pF, n = 14/16; 1 Hz: 1.3 ± 0.3 pA/pF, n = 11/20; p < 0.0001 One-Way ANOVA). (D)The Na_v_-mediated current was verified by replacing the extracellular Na^+^ concentration with equimolar N-methyl-D-glucamine (0Na^+^/NMDG). This replacement effectively eliminated extracellular Na^+^ while preserving osmotic balance[70][71]. In this case, the inward current was significantly reduced (control: 21.6 ± 4.6 pA/pF; n = 5; 0Na^+^/NMDG: 8.8 ± 0.6 pA/pF; n = 5; p = 0.016, Mann-Whitney test. (left) Representative trace in control (black) and after 0 Na^+^ extracellular solution perfusion (red). (right) Average pool data for all the recorded cells in control and with 0 Na^+^ perfusion. (E) Western blot analysis for stemness-related proteins after the 1 Hz stimulation protocol revealed a significant downregulation of SOX2 (SOX2 control: 9.2 ± 0.4, n = 8; SOX2 TTX: 6.0 ± 0.8, n = 7; p = 0.009 t-test) and NANOG (NANOG control: 2.9 ± 0.3, n = 5; NANOG TTX: 1.1 ± 0.3, n = 5; p = 0.002 t-test). (F) GSCs proliferation rate was significantly reduced compared to the control condition (control: 8.9 ± 0.20 x 105 number of cells at 96 hours, n = 6; 1 Hz + TMZ: 3.92 ± 0.12 x 105 number of cells at 96 hours, n = 3; p < 0.0001, Two-way ANOVA), as well as TMZ alone (TMZ: 5.1 ± 0.13 x 105 number of cells at 96 hours, n = 3; p = 0.0015, unpaired t-test).


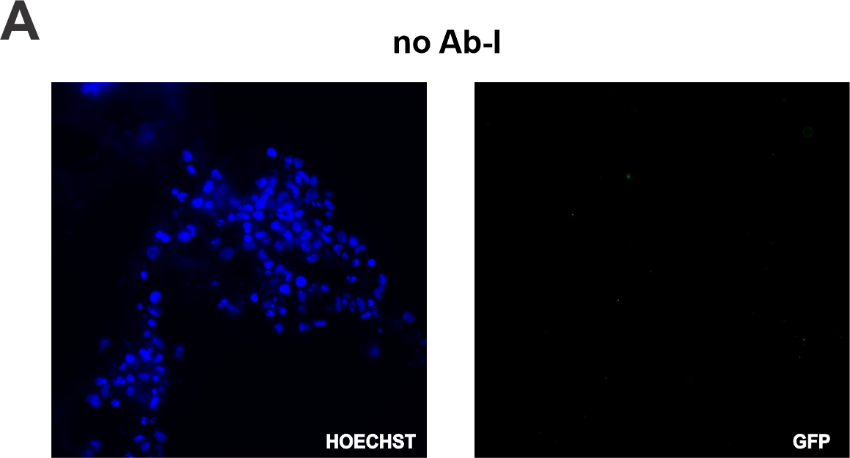


**Supplementary Figure 7 (referring to Fig.6): Characterization of immunoreactivity on 3D GSC cultures and proliferation assay**

1. To assess potential background, a negative control involves incubating the cells with the secondary antibody alone, excluding the primary antibody, to eliminate nonspecific staining.


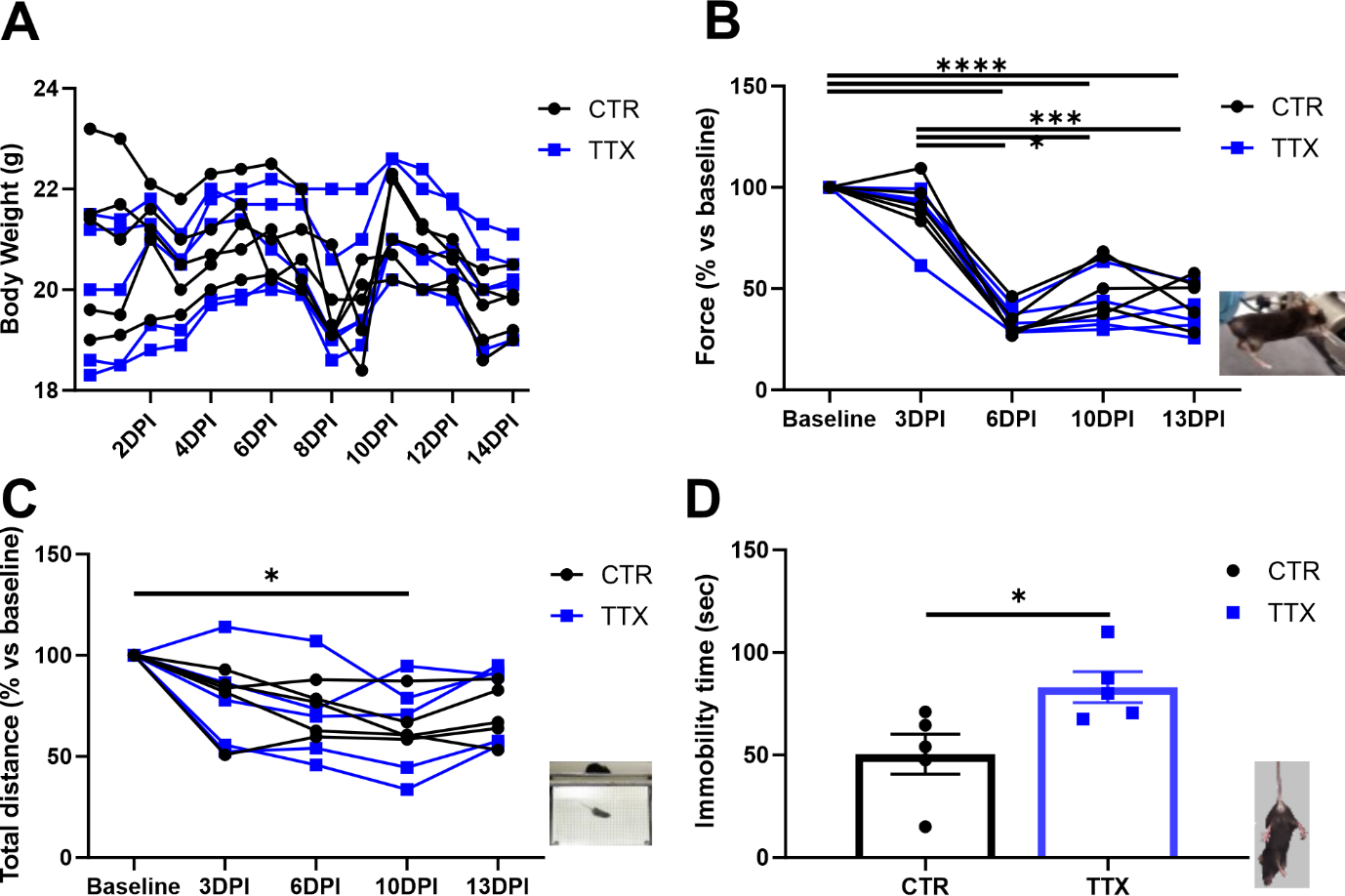


**Supplementary Figure 8 (referring to Fig.7): Assessment of Body Weight, Motor Function, and Behavioral Responses in Control and Tetrodotoxin (TTX)-Treated Glioma Models:** (A) The study compares the mean body weight of control (CTR) and tetrodotoxin (TTX)-treated groups over a 14-day period (Two-way RM ANOVA with Tukey's multiple comparisons test). The analysis reveals no significant differences in body weight between the control and TTX-treated groups across all time points from 0 to 14 days days post murine cancer cells injection (DPI) (Two-way RM ANOVA with Sidak's multiple comparisons test). (B) In the cage mobility test (or grid walk test) both CTR and TTX mice showed a significant lower total distance traveled at 10 DPI respect to the baseline (Two-way RM ANOVA with Tukey's multiple comparisons test, ****P <0.0001). No significant differences were found between CTR and TTX at any time point (Two-way RM ANOVA with Sidak's multiple comparisons test). Interestingly at 13 DPI CTR shows a trend to a motor deficit, while TTX shows motility levels compared to the baseline (CTR Baseline vs 13DPI P = 0.77; TTX Baseline vs 13DPI P = 0.28). (C) In the grip strength test both CTR and TTX mice exerted with their forelimbs an average force significantly lower at 6DPI, 10DPI and 13 DPI respect to the baseline and to 3DPI (Two-way RM ANOVA with Tukey's multiple comparisons test, *P = 0.030). No significant differences were found between CTR and TTX at any time point (Two-way RM ANOVA with Sidak's multiple comparisons test). (D) Tail suspension test shows a higher immobility time in TTX group with respect to the CTR group at 13 DPI (CTR = 50.40 ± 9.74; TTX = 83.10 ± 7.60; unpaired t test, *P = 0.023). All data are expressed as mean ± SEM. * P <0.05, ** P<0.01, *** P <0.001, **** P <0.0001.

**
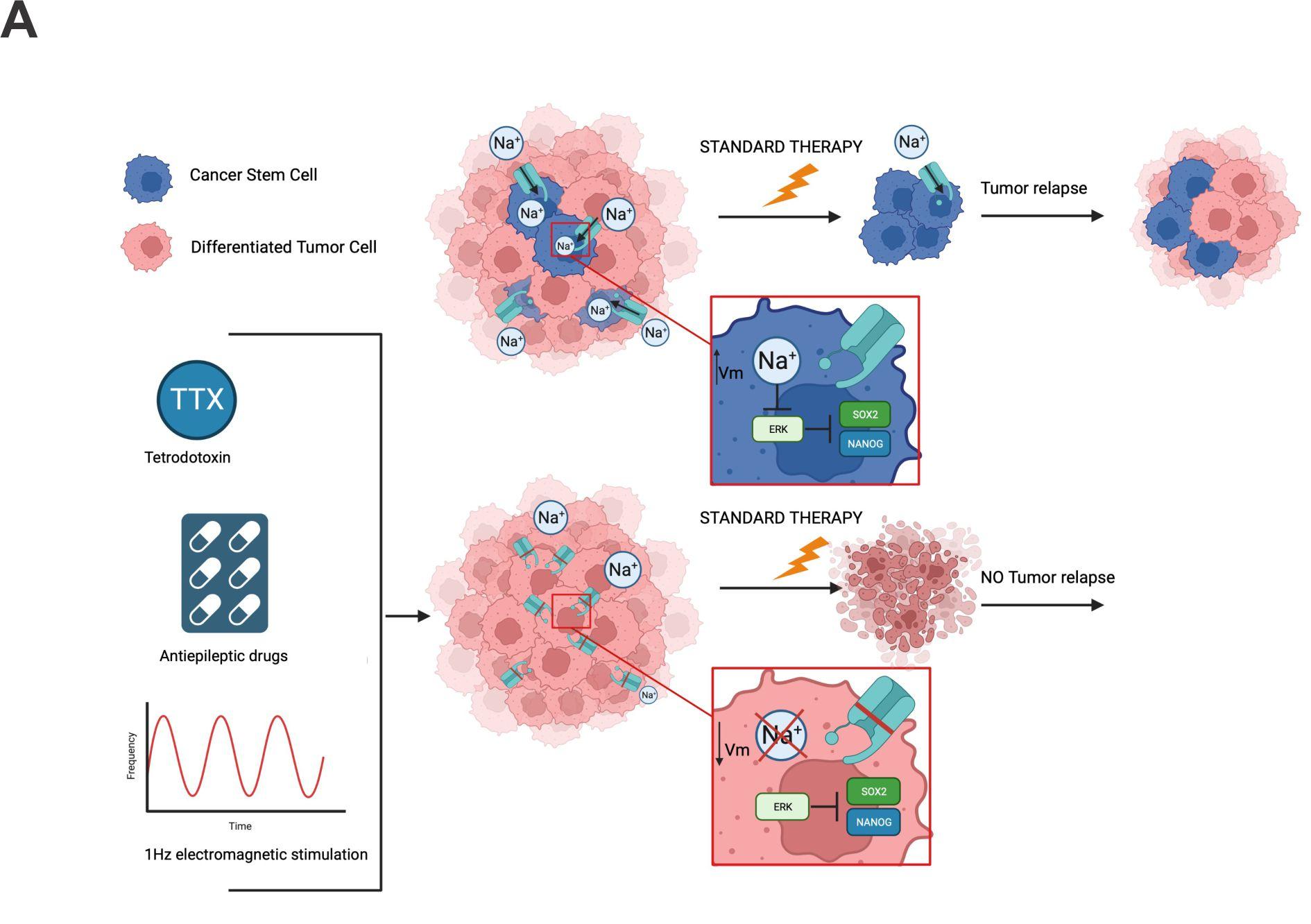
**

**Supplementary Figure 9: Graphical Abstract:** (A) Graphical abstract summarizing the role of Na_v_ in GBM. (top) Na_v_ is associated with stemness markers in GSCs and it is upregulated in response to chemotherapy. The maintenance of stemness mediated by the Na_v_ channel favors the resistance to standard therapy. Blockade or downregulation of Na_v_ results in reduction of stemness-related proteins as well as a significant fraction of GSCs that, from G0 phase, enter in the cell cycle and become sensitive to TMZ. This results in higher chemotherapy efficacy and GSCs loss of relapse capability.
